# Universal probe-based intermediate primer-triggered qPCR (UPIP-qPCR) for SNP genotyping

**DOI:** 10.1186/s12864-021-08148-2

**Published:** 2021-11-24

**Authors:** Baowei Li, Yanran Liu, Xiaodan Hao, Jinhua Dong, Limei Chen, Haimei Li, Wei Wu, Ying Liu, Jianxun Wang, Yin Wang, Peifeng Li

**Affiliations:** 1 Key Laboratory of Biological Medicines in Universities of Shandong Province, Weifang Key Laboratory of Antibody Medicines, School of Bioscience and Technology, Weifang Medical University, Jinan, 261053 Shandong China; 2 Institute for Translational Medicine, Qingdao University, Qingdao, 266021 China; 3 School of Basic Medicine, Qingdao University, Qingdao, 266021 China; 4 College of Food Science and Engineering, Qingdao Agricultural University, Qingdao, 266109 China

**Keywords:** UPIP-qPCR, Intermediate primer, Universal probe, SNP genotyping, Sanger sequencing

## Abstract

**Background:**

The detection and identification of single nucleotide polymorphism (SNP) is essential for determining patient disease susceptibility and the delivery of medicines targeted to the individual. At present, SNP genotyping technology includes Sanger sequencing, TaqMan-probe quantitative polymerase chain reaction (qPCR), amplification-refractory mutation system (ARMS)-PCR, and Kompetitive Allele-Specific PCR (KASP). However, these technologies have some disadvantages: the high cost of development and detection, long and time consuming protocols, and high false positive rates. Focusing on these limitations, we proposed a new SNP detection method named universal probe-based intermediate primer-triggered qPCR (UPIP-qPCR). In this method, only two types of fluorescence-labeled probes were used for SNP genotyping, thus greatly reducing the cost of development and detection for SNP genotyping.

**Results:**

In the amplification process of UPIP-qPCR, unlabeled intermediate primers with template-specific recognition functions could trigger probe hydrolysis and specific signal release. UPIP-qPCR can be used successfully and widely for SNP genotyping. The sensitivity of UPIP-qPCR in SNP genotyping was 0.01 ng, the call rate was more than 99.1%, and the accuracy was more than 99.9%. High-throughput DNA microarrays based on intermediate primers can be used for SNP genotyping.

**Conclusion:**

This novel approach is both cost effective and highly accurate; it is a reliable SNP genotyping method that would serve the needs of the clinician in the provision of targeted medicine.

**Supplementary Information:**

The online version contains supplementary material available at 10.1186/s12864-021-08148-2.

## Background

In a clinical setting, single nucleotide polymorphism (SNP) genotyping provides a door into recognizing individual susceptibility to disease and underpins the prospect of targeted medicine [1–4]. Currently, SNP genotyping technology mainly includes first-generation sequencing, TaqMan probe quantitative polymerase chain reaction (qPCR), amplification-refractory mutation system (ARMS)-PCR, and Kompetitive Allele-Specific PCR (KASP [5–7]. However, these technologies have limitations such as high cost of development and detection, long and time consuming protocols, and high false positive rates [5, 8].

Sanger sequencing is very accurate and could be considered the gold standard for the identification and detection of SNPs [9, 10]. However, Sanger sequencing requires expensive equipment, running costs (for example reagent costs) are high, and workflows are laborious and slow; consequently, Sanger sequencing is not well suited to a clinical setting. TaqMan probe-based qPCR, the most widely used SNP detection method in clinical diagnostics, is rapid, highly accurate and has a low cost per assay. However, probe optimization is a complex and slow process, making marker development expensive. This has limited its application in large-scale SNP genotyping [11–15].

ARMS-PCR is an amplification-refractory mutation system [16, 17]. ARMS-PCR has a tendency to produce false positives when the number of cycles of amplification is high, thus significantly limiting the application of this method in clinical detections [18].

Universal template probe assays based on qPCR [19] and KASP [20], unlike TaqMan, lack specific-sequence primers and usually result in false-positive results, which makes them difficult to use for clinical examinations [21]. SNP arrays are able to detect large numbers of SNPs in a single assay but they are expensive and protocols are long and laborious, which making it unsuitable for clinical individualized SNP genotyping [5, 22].

In view of the limitations of the above technologies and the actual needs of clinical SNP detection, we established a new and improved SNP detection method, named universal probe-based intermediate primer-triggered qPCR (UPIP-qPCR). In the development of any type of SNP genotyping kit based on this method, two types of universal fluorescence probes are used, and intermediate primers are introduced to guarantee specificity, so that the cost and duration of research and development for genotyping kits are significantly reduced, and UPIP-qPCR gives rise to the need for low cost and high accuracy of clinical SNP genotyping.

## Results

### Feasibility verification of UPIP-qPCR in SNP genotyping

**Fig. 1:**
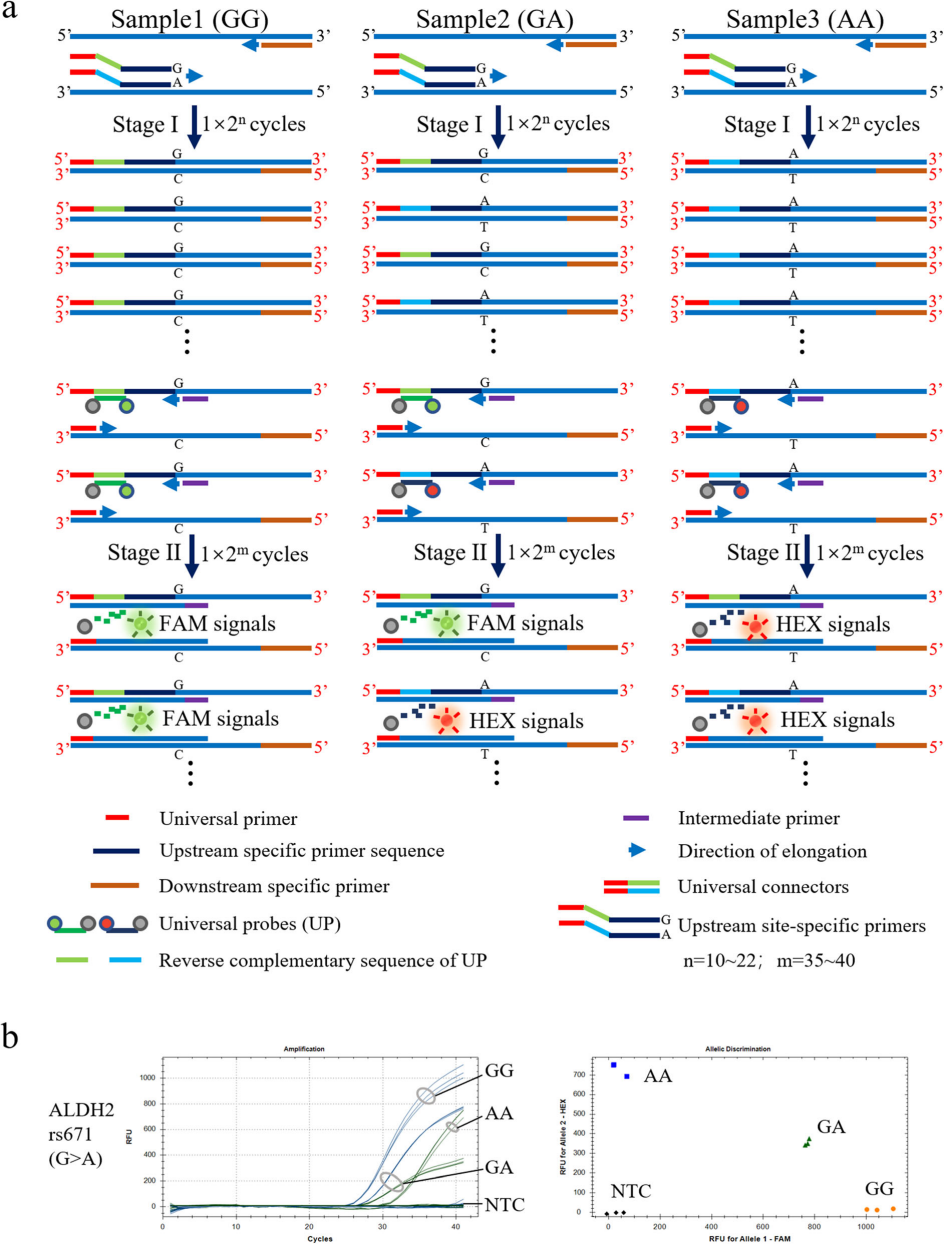
Principle and application of UPIP-qPCR for SNP genotyping. The reactions of UPIP-qPCR are divided into two stages (a). The first stage is a general PCR reaction aiming to obtain a certain amount of DNA fragments containing specific SNP sites (a, upper panel in each sample). The second stage is a quantitative PCR (qPCR) reaction, which uses the PCR products of the first stage as templates to obtain the corresponding fluorescence signals of alleles (a, lower panel in each sample). UPIP-qPCR was initially used to identifying the SNP genotypes of ALDH2 rs671 (b). All three genotypes of each SNP samples successfully yielded accurate results by UPIP-qPCR. The homozygous WT genotypes presented by FAM-only signals (blue curves) were shown in the lower right area of the genotyping scatter diagrams with yellow dots, the heterozygous genotypes presented by FAM and HEX signals (blue curves and green curves) were shown in the middle area of the genotyping scatter diagrams with green triangles, and the homozygous mutant genotypes presented by HEX-only signals (green curves) were shown in the upper left area of the genotyping scatter diagrams with blue squares. No-template control groups (NTC) were shown in the lower left area of the genotyping scatter diagrams with black rhombuses. Each sample was detected with three duplicates in one experiment, and the experiments were repeated more than three times

Universal probe-based and intermediate primer-triggered qPCR (UPIP-qPCR) is a two-step process. The first step, a standard PCR reaction, takes c. 30 mins. The second step, the qPCR reaction which uses the product of the first step, takes c. 60 mins, to obtain the corresponding fluorescence signals of the alleles (Fig. 1a and Fig. S1a).

We used rs671 and rs2031920 loci as candidate SNPs to verify the feasibility of UPIP-qPCR and optimize this technology. Following the reaction, the corresponding genotypes were identified according to the final fluorescence category and intensity (relative fluorescence units, RFU). The results showed a typical S-type amplification curve with exponential growth and the amplification signals of the three positive DNA standards were specific. The amplification signals of the GG genotype were only FAM-positive, the GA genotype was both FAM- and HEX- positive, the AA genotype was only HEX-positive, and the no-template control group (NTC) had no false-positive signals, indicating the accuracy of the genotyping results (Fig. 1b and Fig. S1b). These results indicate that UPIP-qPCR is feasible for SNPs genotyping. As a new genotyping method, its time and cost consumption are less than those of TaqMan probe-qPCR and Sanger sequencing, and its operation complexity is moderate (Table 1). Thermal cycles, primer concentrations and products dilution ratios of the first stage play important roles in the UPIP-qPCR, and the PCR products should be diluted 10 times before used in the second stage (Fig. S2).

**Table 1:** Characteristics of Three Methods for SNP Genotyping

	UPIP-qPCR	TaqMan	Sanger
Reagent cost for kit development ($/SNP)	35–70	500–1000	10–20
Reagent cost for detection ($/SNP)	~ 0.3	~ 1	~ 3
Detection period (hours/SNP)	1.5–2	1.5–2	12–24
Operation complexity	Moderate (Two stages)	Low (One stages)	High (Five stages)

### UPIP-qPCR presented high sensitivity in SNP genotyping detections

**Fig. 2:**
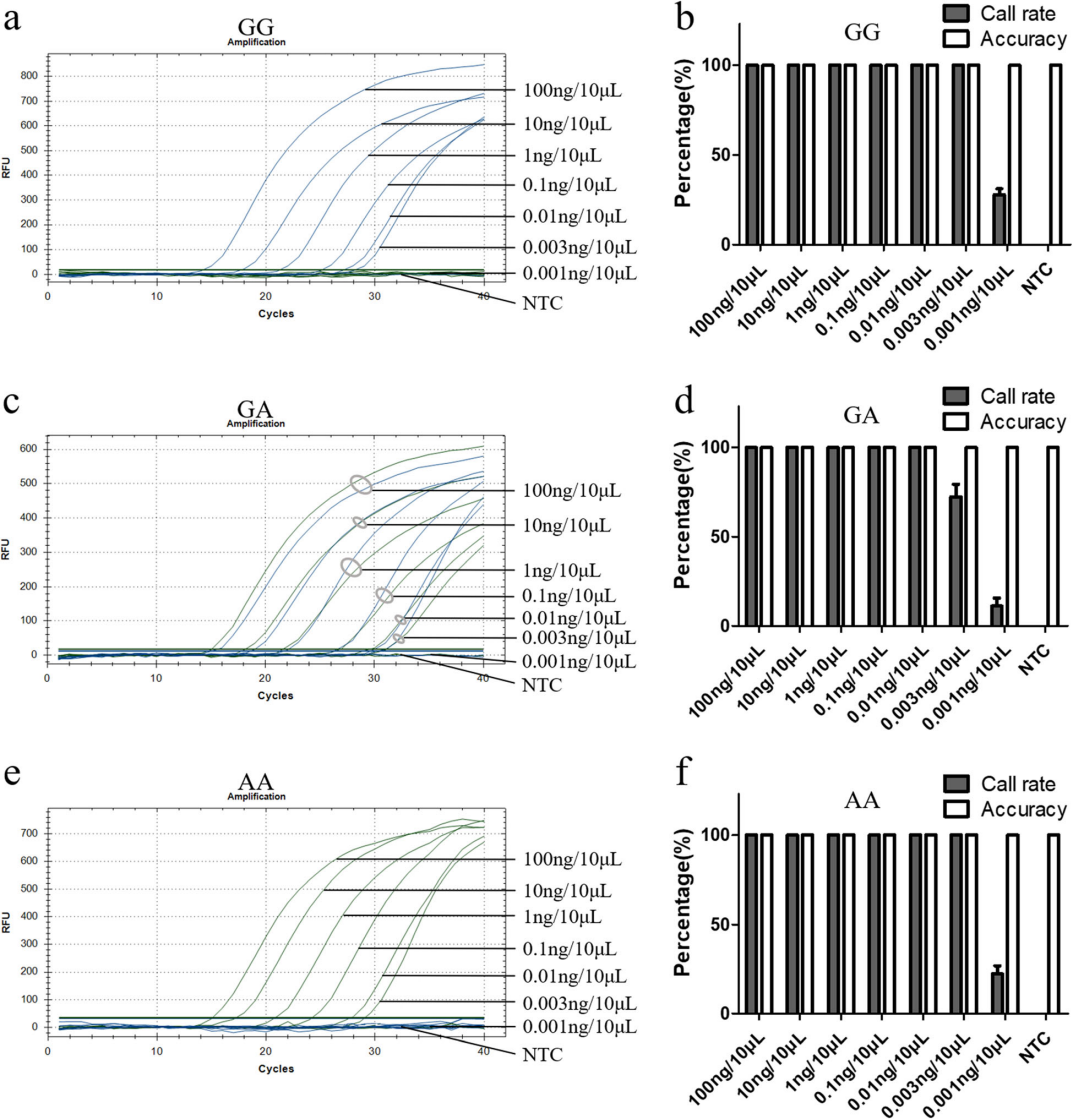
Sensitivity analysis of UPIP-qPCR. Three genotypic DNA samples of ALDH2 rs671 with concentrations from 100 ng/10 μL to 0.003 ng/10 μL showed typical S-type curves, and the appearance order of the curves was positively correlated with the decrease in concentration gradient (a, c and e). The call rates were 100% in all three genotypic samples with concentrations from 100 ng/10 μL to 0.01 ng/10 μL, but < 100% in concentrations of 0.003 ng/10μLand 0.001 ng/10 μL (b, d and f). The accuracy of all three genotypic genomic DNA concentrations was 100% (b, d and f). FAM and HEX signal curves of the same concentration in genotype GA were indicated by a gray ellipse. Each sample was detected with nine duplicates in one reaction, and the experiments were repeated four times. Bars show *SD* (*n* = 36)

The sensitivity of UPIP-qPCR was analyzed using a primer concentration of 100 nM/each and 18 cycles of amplification in the first-stage reaction; human genomic DNA samples of three genotypes of rs671 with different concentrations were used. The results showed that most of the DNA samples with different concentrations presented typical S-type curves, and the order of appearance of the curves of amplification (S-shaped curves) was inversely related to the concentration of DNA in the samples (Fig. 2a, c and e). The accuracies of all the concentrations of three genotypic genomic DNA were 100%, and although there were good call rates in the high concentration samples, it was not ideal in the low concentration samples (Fig. 2b, d and f). Specifically, the call rates of all three genotypes were 100% at five concentration gradients from 100 ng/10 μL to 0.01 ng/10 μL, and the copy number gradients from 33,000 genomic DNA per 10 μL to 3 genomic DNA per 10 μL. The other call rates were: 100% for GG and AA samples with a concentration of 0.003 ng/10 μL, 72.22% for GA samples with a concentration of 0.003 ng/10 μL, 27.78, 11.11 and 22.22% for GG, GA and AA samples with a concentration of 0.001 ng/10 μL, respectively (Fig. 2b, d and f). The above data showed that the concentration of 0.01 ng/10 μL has the highest sensitivity of UPIP-qPCR, i.e., every 10 μL reaction system containing three copies of genomic DNA can obtain reliable genotyping results.

TaqMan probe-qPCR was performed to compare the sensitivity of the two methods. The results showed that DNA samples with high concentrations presented typical S-type curves, (Fig. S4a, S4c and S4e). The accuracies in three genotypic genomic DNA with concentrations from 100 ng/10 μL to 0.01 ng/10 μL were 100% (Fig. S4b, S4d and S4f). Call rates in three genotypic genomic DNA with concentrations from 100 ng/10 μL to 0.1 ng/10 μL were100%, but were < 100% or even 0% in concentrations from 0.03 ng/10 μL to 0.01 ng/10 μL (Fig. S4b, S4d and S4f). These data showed that the concentration of 0.1 ng/10 μL was the highest sensitivity of TaqMan probe-qPCR, i.e., every 10 μL reaction system containing 33 copies of genomic DNA can obtain reliable genotyping results by TaqMan probe-qPCR method.

### UPIP-qPCR possessed high call rate and accuracy

In this study, the genotypes of rs671, rs1057910, rs9923231, rs1801131, rs1801133, and rs1801394 in 224 DNA samples were detected by UPIP-qPCR, TaqMan probe-qPCR, KASP and Sanger sequencing. The UPIP-qPCR scatter plots showed good signal differentiation. Each scatter diagram contained three repeats of positive standards and NTC. The yellow dots represent the wild-type genotype, the green triangles represent the heterozygous genotype, and the blue squares represent the mutant genotype (Fig. S5a). The TaqMan probe-qPCR scatter plots also showed good signal differentiation in the genotyping detection of these six SNPs (Fig. S5b). The KASP scatter plots showed many undefined dots (Fig. S5c). The call rates of rs671, rs1057910, rs9923231, rs1801131, rs1801133, and rs1801394 generated by UPIP-qPCR were 99.11, 100, 100, 100, 99.55 and 100% respectively, which were all higher than those of TaqMan probe-qPCR and KASP (Table 2). Compared with Sanger sequencing results (Additional file 3), the accuracies of UPIP-qPCR and TaqMan probe-qPCR were all 100% in the detection of these six SNPs, and the accuracies of KASP were all below 100% (Table 2). By counting the allele frequency, the minor allele frequency (MAF) of 224 samples detected by UPIP-qPCR was similar to MAF values of East Asians derived from ALFA or 1000 Genomes (NCBI) (Table S7), which indicated that UPIP-qPCR has sufficient ability to recognize the distribution of SNP genotypes in a specific human population.

**Table 2:** Call Rate and Accuracy of UPIP-qPCR, TaqMan Probe-qPCR and KASP

SNPs	Call rate (*n* = 224)			Accuracy (*n* = 224)		
	UPIP-qPCR	TaqMan	KASP	UPIP-qPCR	TaqMan	KASP
rs671	99.11%	98.21%	79.46%	100.00%	100.00%	94.94%
rs1057910	100.00%	98.66%	75.45%	100.00%	100.00%	97.63%
rs9923231	100.00%	97.77%	83.48%	100.00%	100.00%	92.51%
rs1801131	100.00%	97.32%	88.39%	100.00%	100.00%	96.46%
rs1801133	99.55%	95.09%	73.66%	100.00%	100.00%	93.94%
rs1801394	100.00%	96.88%	89.73%	100.00%	100.00%	94.53%

### UPIP-qPCR recognizes all SNP variants and InDels

**Fig. 3:**
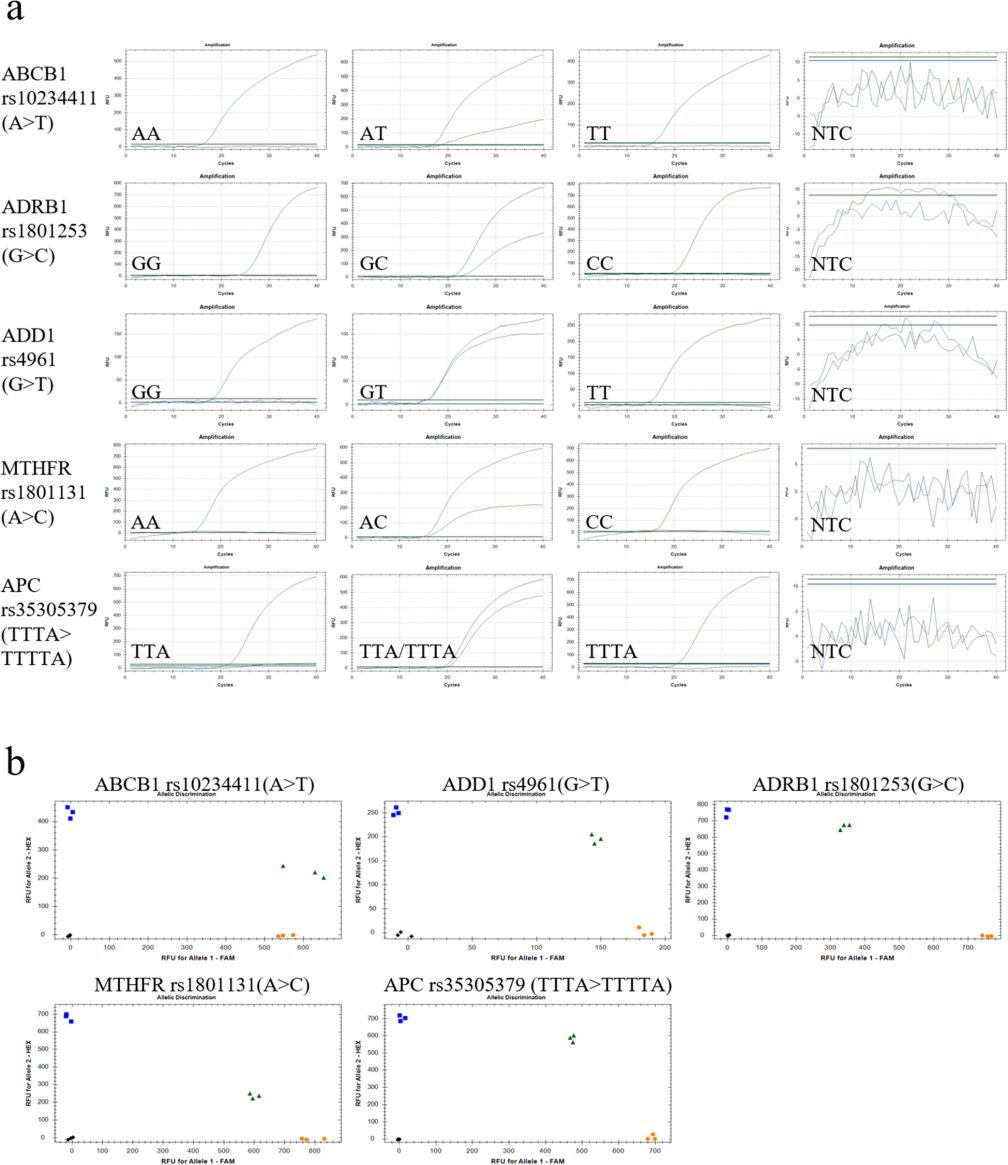
UPIP-qPCR possessed wide applicability in identifying all kinds of variations in SNPs. UPIP-qPCR presented specific amplification signals and typical S-type curves in the genotype detection of 5 different SNPs with all kinds of variations except G-A and C-T, including interchanges between A-C, A-T, G-C, G-T,, and base InDels mutation (a). All three different genotypes of these SNPs were clustered in the scatter diagrams, and the results of genotyping were correct compared to Sanger sequencing(b). In each scatter diagram, WT-, heterogeneous-, and mutant-genotypes were represented by yellow dots, green triangles and blue squares, respectively. Each sample was detected with three duplicates at one experiment, and the experiments were repeated more than three times

There are six types of nucleotide alterations in point mutations, including interchanges between A-G, A-C, A-T, G-C, G-T and C-T. InDels are defined as the insertion or a deletion of one or more nucleotides into a DNA sequence with respect to a defined reference sequence. In addition to the A-G, A-C and C-T mutation types involved in the above experiments, we also selected other SNPs of all mutation types to test the wide adaptability of UPIP-qPCR. The genes and SNP sites were as follows: *ABCB1* rs10234411 (A > T), *ADD1* rs4961 (G > T), *ADRB1* rs1801253 (G > C), *MTHFR* rs1801131 (A > C), *MTHFR* rs1801133 (C > T), *MTRR* rs1801394 (A > G), *ABCB1* rs1045642 (T > C), *DPYD* rs3918290 (G > A), *DPYD* rs55886062 (A > C), *GSTP1* rs1695 (A > G), *XRCC1* rs25487 (A > G), *APC* rs35305379 (TTTA > TTTTA), *APC* rs34481414 (ACTACAAT > ACAAT). These SNPs are of significance in determining individual responses to medical treatments. The results showed that UPIP-qPCR was able to identify all SNP types regardless of whether they were transitions of transversions (Fig. 3a and Fig. S6a). The clustering of fluorescent signals for the homozygous and heterozygous calls was distinct and clear in the scatter diagrams (Fig. 3b and Fig. S6b), and the results were accurate. These results suggests that UPIP-qPCR can be widely used to genotype different SNPs and InDels.

### Microarrays based on intermediate primers were feasible for SNP genotyping

**Fig. 4:**
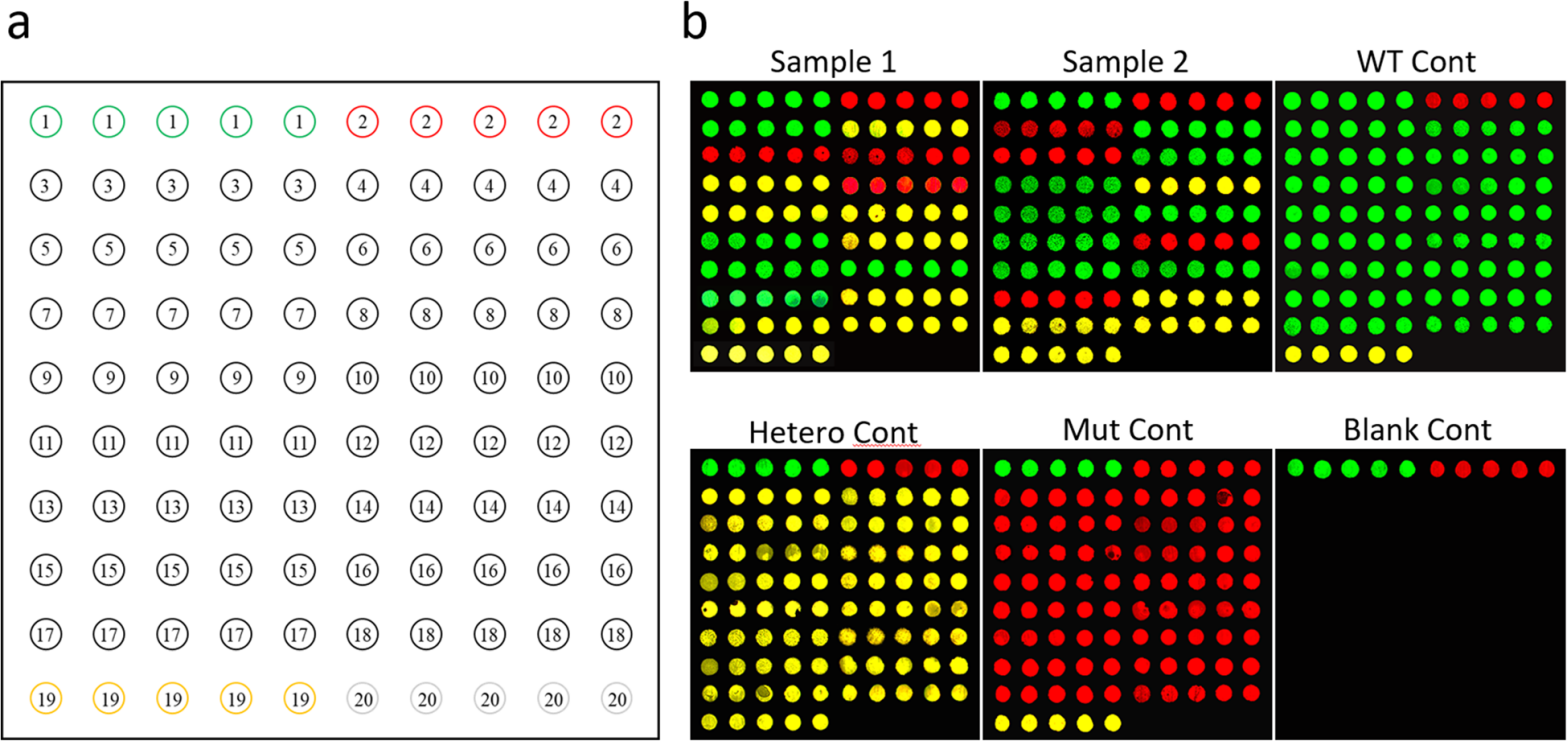
Microarrays for genotyping. Microarrays were made of 20 types of probes with five repeated dots, the order of probes was marked by circled numbers, and the SNPs and sequences related to these probes were listed in Table S9 with the same No., which briefly, are ① FAM positive control probes, ② HEX positive controls, ③ ~ ⑱ probes of 16 kinds of SNPs, ⑲ internal control GAPDH probes, ⑳ negative control NH_2_-dT_14_ probes (a). The hybridization signals of the two types of human genomic DNA (No.1 and No.2) were both specific and correct (b). The positive control DNA was able to obtain specific and accurate hybridization signals. The hybridization signals of wild-type DNA were FAM-positive and HEX-negative (green dots), the hybridization signals of heterozygous DNA were FAM- and HEX- double-positive (yellow dots), and the hybridization signals of mutant DNA were FAM-negative and HEX-positive (red dots) (b). There were no signals on the blank control microarray with water as template, except for FAM- and HEX- positive control probes (b). FAM (green), HEX (red), FAM and HEX (yellow) and no signal (black background) were displayed respectively at the positions of ①, ②, ⑲and⑳ (b). WT: wild type; Mut: mutant; Hetero: Heterozygous; Cont: control

As the intermediate primers were able to recognize the template DNA specifically, 20 × 5 dot DNA microarrays were made (Fig. 4a) with 16 types of SNP intermediate primers, positive primers, negative primers, and internal reference (GAPDH) primers as probes, and the hybridization method was utilized to achieve SNP genotyping. Using multiplex PCR, and single-labeled fluorescent probes plus universal reverse primer-PCR, we prepared DNA templates for hybridization, and adopted an overnight hybridization method (Fig. S7, Table S8 and Table S10). The results showed that the positive reference DNA of the three genotypes could obtain specific and accurate hybridization signals. The hybridization signals of homozygous wild-type, heterozygous, and homozygous mutant DNA were FAM-positive and HEX-negative (green dots), FAM- and HEX-positive (yellow dots), and FAM-negative and HEX-positive (red dots), respectively. (Fig. 4b). There were no signals on the blank control microarray that used water as a template, except for the FAM- and HEX-positive control dots (Fig. 4b). FAM (green), HEX (red), FAM and HEX (yellow) and no signal (black background) were displayed at the positions of FAM-positive, HEX-positive, GAPDH and negative reference probes fixed on each microarray, and were consistent with the expected results (Fig. 4b). The hybridization signals of the two types of human genomic DNA (No. 1 and No. 2) were also specific, and consistent with the Sanger sequencing results (Fig. 4b, Table S11, and Additional file 3). Sixteen SNPs can be genotyped for one DNA sample by one microarray simultaneously using this method, thus increasing the detection throughput.

Please see “Additional file 2“to get more information about results of this study.

## Discussion

In the UPIP-qPCR, intermediate primers were designed to ensure the accuracy of the signal. This was because these primers would only combine with the template DNA to generate specific signals, when the products of the first stage were of the correct DNA segments. In principle, the closer the 3′-end of the intermediate primer to the SNP locus, the higher the amplification efficiency; therefore, we suggest that the intermediate primers should be designed ≤30 bases from their 3′-end to the SNP loci (Fig. S3 and Table S4).

Although two-stage reactions were required, the sensitivity of UPIP-qPCR reached three copies per 10 μL reaction system, which could compensate this operational defect. Call rates were close to 100%, but this high figure was dependent on the quality of the DNA used in the assay; it should be of high purity. Indeed, the call rate and accuracy of UPIP-qPCR were higher than those of TaqMan probe-qPCR (the standard assay presently in use) and KASP. Other cost-effective, genotyping tools proved to be less accurate and to have lower call rates than TaqMan, such as high-resolution melting (HRM) technology. For this reason, these assays were not directly included in this study [23, 24]. NGS technology is characterized by high-throughput sequencing, and can detect millions of DNA sequences simultaneously. It has the advantages of massive unknow-gene mutation screening and massive SNP genotyping. However, NGS technology is not suitable for and seldom used in genotyping small numbers of known SNPs, owing the long reaction period, high cost of reagents and expensive machines [25, 26]. There is no comparability between UPIP-qPCR and NGS, because they have specific application fields in SNP genotyping, the former for seldom known SNPs, and the latter for massive unknowns.

The first stage of UPIP-qPCR can be performed on a single target or multiplexed depending on the requirements of the clinician. A single PCR can be adopted when the number of SNPs detected is low and the amount of DNA available is sufficient; otherwis, multiplex PCR can be adopted when the number of SNPs detected is high, and the quantity of DNA available is limited.

The microarray hybridization experiment with intermediate primers as probes provided a basis for developing a new SNP screening method with higher throughput and high accuracy, which would further reduce the price of SNP genotyping. In addition, intermediate primers can also be fixed in multi-hole fluorescence quantitative microfluidic reaction plates, such as Thermofisher QuantStudio 12 K. In this case, only the universal primer, universal probes and stage I multiplex PCR products need to be added to the reaction system, so that in addition to high-throughput SNP genotyping, high-throughput detection of copy number variation (CNV) can also be carried out, thus extending the application range of UPIP-qPCR. Although, we are looking forward to developing a high-throughput real-time planar fluorescent qPCR and hybridization-sequencing technology based on intermediate primers, achieving accurate results and analysis would be a challenging task.

## Conclusions

In this study, we successfully developed an efficient and cost-effective method for SNP genotyping, called universal probe-based intermediate primer-triggered qPCR (UPIP-qPCR). The sensitivity of UPIP-qPCR in SNP genotyping was 0.01 ng, the call rate was more than 99.1%, and the accuracy was more than 99.9%, which were both higher than those of TaqMan probe-qPCR. UPIP-qPCR can recognize all type of SNP variants (interchanges between A-G, A-C, A-T, G-C, G-T or C-T) and InDels. Microarrays based on intermediate primers were feasible for SNP genotyping, which may promote a novel high-throughput SNP genotyping method in the future.

In summary, the UPIP-qPCR developed in this study is a novel SNP genotyping technology with low cost, fast detection, and high accuracy, which will help to reduce the cost of clinical detection, reduce the burden of patients, and promote the development of precision medicine.

## Methods

### PCR and Sanger sequencing

The nucleotide sequences containing specific SNPs were acquired from the dbSNP database of the National Center for Biotechnology Information (NCBI, Bethesda, MD, USA) (https://www.ncbi.nlm.nih.gov/snp/). The primers flanking the SNP site (Fla-primers) and sequencing primers (Seq-primers) were designed using Primer Premier version 5 software (Table S1). Polymerase chain reactions (PCR) were performed to amplify 151–921 bp products containing the targeted SNPs. The nucleotide sequences of the PCR products containing specific SNPs were obtained using a 3730xl DNA analyzer (Applied Biosystems). All blood samples were collected at the Affiliated Yantai Yuhuangding Hospital of Qingdao University. Genomic DNA was isolated from peripheral whole blood samples using the Gentra Puregene Blood Kit (Qiagen Corp, CA) according to the manufacturer’s instructions.

### UPIP-qPCR

The UPIP-qPCR consisted of two separate reactions. The first stage (stage I) of UPIP-qPCR was a general PCR. The reaction conditions were as follows: initial denaturation at 95 °C for 3 min, followed by 10–22 cycles of denaturation at 98 °C for 5 s, annealing at 67 °C for 25 s, elongation at 72 °C for 20 s, and final elongation at 72 °C for 1 min. PCR products were diluted ten times with ddH_2_O and used as templates in the second-stage reaction.

The second stage (stage II) of UPIP-qPCR was performed using a qPCR. The reaction conditions were as follows: initial denaturation at 95 °C for 3 min, followed by 35–40 cycles of denaturation at 98 °C for 5 s, annealing at 49 °C for 25 s, elongation at 72 °C for 1 s and fluorescent signals were obtained via plate reading. Sequences of primers used for UPIP-qPCR are listed in Table S2. Sequences of FAM- and HEX-labeled universal probes and universal primers are listed in Table S3.

### Feasibility verification of UPIP-qPCR

For this part, we designed the Sanger sequencing related primers (Table S1) for rs671 and rs2031920, through Sanger sequencing to obtain homozygous wild-, heterozygous, and homozygous mutant- types of human genome DNA positive standards. A pair of upstream site-specific primers were designed (Table S2). The first stage of the reaction was carried out on an ordinary PCR instrument, with a total of 10 cycles, at an annealing temperature of 67 °C, to complete the initial amplification of DNA fragments. In the second stage, the signals were collected in real-time as the reaction was carried out on a qPCR instrument with 40 cycles, at an annealing temperature of 49 °C.

### Sensitivity analysis

The genomic DNA samples of the three genotypes of ALDH2 rs671 were used for UPIP-qPCR sensitivity analysis, and ddH_2_O was used for NTC. In the first stage of UPIP-qPCR, the concentration gradients of genomic DNA were 100 ng, 10 ng, 1 ng, 0.1 ng, 0.01 ng, 0.003 ng and 0.001 ng per 10 μL reaction system. TaqMan probe-qPCR was set as control method for the sensitivity analysis. The accuracies mentioned in this section indicates the reproducibility of results when the assay is repeated using the same DNA concentration. Sequences of primers and probes for TaqMan probe-qPCR are listed in Table S5.

### Analysis of call rate and accuracy for UPIP-qPCR

UPIP-qPCR was used to detect the genotypes of 224 human genomic DNA samples at the rs671, rs1057910, rs9923231, rs1801131, rs1801133, and rs1801394 loci. The reaction system and detailed thermal cycle parameters can be found in “UPIP-qPCR”. The number of effective results with the total number of samples were compared to obtain the call rate of the UPIP-qPCR method, and the genotyping results were compared with the Sanger sequencing results of the same samples to obtain the accuracy rate of the UPIP-qPCR method. TaqMan probe-qPCR and KASP were used as control methods for the analysis of the call rate and accuracy. Sequences of primers and probes for TaqMan probe-qPCR and KASP are listed in Table S5 and S6, respectively.

### Wide applicability analysis of UPIP-qPCR in SNP genotyping

Based on UPIP-qPCR, we designed primers (Table S2) and genotyped 13 different SNPs. These SNPs covered all SNP mutation types. The system volumes of the first and second-stage UPIP-qPCR reactions were all 10 μL, and the cycle numbers of the first and second-stage reactions were 10 and 40, respectively. Please refer to “UPIP-qPCR” for the reagent composition of the system.

### SNP microarray assay based on intermediate primers

The microarrays integrated 20 types of probes with five duplicates, including these probes included intermediate primers of 16 types of SNPs, complementary sequences of FAM and HEX single-labeled primers as a positive reference, intermediate primers of GAPDH as an internal reference, and amino modified 14-poly deoxythymine (NH_2_-dT_14_) as negative control probes. See Table S9 for the sequences of the probes.

The preparation process of the PCR products for hybridization was divided into three stages. The first stage was multiplex PCR. In the first stage, normal human genome DNA (No. 1 and No. 2) was used as the template DNA of the experimental group, and the template DNA of the control groups was divided into three types, namely, the mixture of wild-type, heterozygous and mutant positive control DNA of 16 SNP sites, and the wild type positive control DNA of GAPDH. ddH_2_O was used as the blank control template. The second stage was a product treatment process, that is, using exonuclease I to digest the products of the first stage which would be used as templates in the third stage reactions. The third-stage was the fluorescence labeling PCR reaction. In the third stage, FAM and HEX single-labeled primers and universal reverse primer (Table S3) were combined to amplify the templates to obtain sufficient DNA fragments for microarray hybridization.

Products of the third stage were denatured at 95 °C for 5 min, cooled on ice for 2 min, and then mixed with an equal volume hybridizing buffer to form a hybridizing solution. Hybridizing solution (20 μL) was aliquoted into the microarrays, covered with coverslips, and incubated for 16–20 h. Images of the FAM and HEX signals were captured using a confocal microscope. Dots with only FAM signals (green) denoted the homozygous wild type, those with only HEX signals (red) were homozygous mutants, and those with both signals (yellow) were heterozygous.

### Data analyses

One-way ANOVA tests were used for data comparation between the groups. Differences were considered statistically significant when *P* < 0.05. Data analyses were performed using SPSS 16.0.

Please see “Additional file 1“to get more detailed methods.

## Supplementary Information

**Additional file 1.** Detailed Materials and Methods for UPIP-qPCR.

**Additional file 2.** Additional results of UPIP-qPCR.

**Additional file 3.** GenBank accession numbers.

**Additional file 4.** Supplementary figure legends.

**Additional file 5.** Supplementary Tables.

## Data Availability

The datasets supporting the conclusions of this article are available in the NCBI GenBank repository with accession number OK351361 - OK352256, OK480620 - OK480877, OK492234 - OK492457, https://www.ncbi.nlm.nih.gov/nuccore/. Definitions for these accession numbers can be found in Additional file 3.

## Abbreviations

UPIP-qPCR: Universal probe-based intermediate primer-triggered qPCR
SNP: Single nucleotide polymorphism
ARMS: Amplification-refractory mutation system
KASP: Kompetitive allele-specific PCR
RFU: Relative fluorescence units
NTC: No-template control
HRM: High-resolution melting
NGS: Next-generation sequencing
CNV: Copy number variation

## References

[1] Scheen AJ (2016). Precision medicine: the future in diabetes care?. Diabetes Res Clin Pract.

[2] Ho DSW, Schierding W, Wake M, Saffery R, O'Sullivan J (2019). Machine learning SNP based prediction for precision medicine. Front Genet.

[3] Tanner JA, Tyndale RF (2017). Variation in CYP2A6 Activity and Personalized Medicine. J Personalized Med.

[4] Li B, Wang X, Hao X, Liu Y, Wang Y, Shan C, Ao X, Liu Y, Bao H, Li P (2020). A novel c.2179T>C mutation blocked the intracellular transport of PHEX protein and caused X-linked hypophosphatemic rickets in a Chinese family. Mol Genet Genomic Med.

[5] Perkel J (2008). SNP genotyping: six technologies that keyed a revolution. Nat Methods.

[6] Matsuda K (2017). PCR-based detection methods for single-nucleotide polymorphism or mutation: real-time PCR and its substantial contribution toward technological refinement. Adv Clin Chem.

[7] You Q, Yang X, Peng Z, Xu L, Wang J (2018). Development and applications of a high throughput genotyping tool for Polyploid crops: single nucleotide polymorphism (SNP) Array. Front Plant Sci.

[8] Kim S, Misra A (2007). SNP genotyping: technologies and biomedical applications. Annu Rev Biomed Eng.

[9] Sanger F, Nicklen S, Coulson AR (1977). DNA sequencing with chain-terminating inhibitors. Proc Natl Acad Sci U S A.

[10] Wu H, Wu X, Liang Z (2017). Impact of germline and somatic BRCA1/2 mutations: tumor spectrum and detection platforms. Gene Ther.

[11] Lee LG, Connell CR, Bloch W (1993). Allelic discrimination by nick-translation PCR with fluorogenic probes. Nucleic Acids Res.

[12] Dooms M, Chango A (2014). Abdel-nour a: [quantitative PCR (qPCR) and the guide to good practices MIQE: adapting and relevance in the clinical biology context]. Ann Biol Clin.

[13] Chu MKM, Wong EYL, Wong SCC, Alikian M, Gale RP, Apperley JF, Foroni L (2017). Molecular techniques for the personalised management of patients with chronic myeloid leukaemia. Expert Rev Mol Diagn.

[14] Sanjuan-Jimenez R, Colmenero JD, Morata P (2017). Lessons learned with molecular methods targeting the BCSP-31 membrane protein for diagnosis of human brucellosis. Clin Chimica Acta Int J Clin Chem.

[15] Zhang L, Cui G, Li Z, Wang H, Ding H, Wang DW (2013). Comparison of high-resolution melting analysis, TaqMan allelic discrimination assay, and sanger sequencing for Clopidogrel efficacy genotyping in routine molecular diagnostics. J Mol Diagnostics JMD.

[16] Newton CR, Graham A, Heptinstall LE, Powell SJ, Summers C, Kalsheker N, Smith JC, Markham AF (1989). Analysis of any point mutation in DNA. The amplification refractory mutation system (ARMS). Nucleic Acids Res.

[17] Newton CR, Heptinstall LE, Summers C, Super M, Schwarz M, Anwar R, Graham A, Smith JC, Markham AF (1989). Amplification refractory mutation system for prenatal diagnosis and carrier assessment in cystic fibrosis. Lancet.

[18] Ferrie RM, Schwarz MJ, Robertson NH, Vaudin S, Super M, Malone G, Little S (1992). Development, multiplexing, and application of ARMS tests for common mutations in the CFTR gene. Am J Hum Genet.

[19] Zhang Y, Zhang D, Li W, Chen J, Peng Y, Cao W (2003). A novel real-time quantitative PCR method using attached universal template probe. Nucleic Acids Res.

[20] He C, Holme J, Anthony J (2014). SNP genotyping: the KASP assay. Methods Mol Biol (Clifton, NJ).

[21] Ertiro BT, Ogugo V, Worku M, Das B, Olsen M, Labuschagne M, Semagn K (2015). Comparison of Kompetitive allele specific PCR (KASP) and genotyping by sequencing (GBS) for quality control analysis in maize. BMC Genomics.

[22] Ragoussis J (2006). Genotyping technologies for all. Drug Discov Today Technol.

[23] Gundry CN, Vandersteen JG, Reed GH, Pryor RJ, Chen J, Wittwer CT (2003). Amplicon melting analysis with labeled primers: a closed-tube method for differentiating homozygotes and heterozygotes. Clin Chem.

[24] Martino A, Mancuso T, Rossi AM (2010). Application of high-resolution melting to large-scale, high-throughput SNP genotyping: a comparison with the TaqMan method. J Biomol Screen.

[25] Cirulli ET, Lasseigne BN, Petrovski S, Sapp PC, Dion PA, Leblond CS, Couthouis J, Lu YF, Wang Q, Krueger BJ, Ren Z, Keebler J, Han Y, Levy SE, Boone BE, Wimbish JR, Waite LL, Jones AL, Carulli JP, Day-Williams AG, Staropoli JF, Xin WW, Chesi A, Raphael AR, McKenna-Yasek D, Cady J, Vianney de Jong JMB, Kenna KP, Smith BN, Topp S, Miller J, Gkazi A, al-Chalabi A, van den Berg LH, Veldink J, Silani V, Ticozzi N, Shaw CE, Baloh RH, Appel S, Simpson E, Lagier-Tourenne C, Pulst SM, Gibson S, Trojanowski JQ, Elman L, McCluskey L, Grossman M, Shneider NA, Chung WK, Ravits JM, Glass JD, Sims KB, van Deerlin VM, Maniatis T, Hayes SD, Ordureau A, Swarup S, Landers J, Baas F, Allen AS, Bedlack RS, Harper JW, Gitler AD, Rouleau GA, Brown R, Harms MB, Cooper GM, Harris T, Myers RM, Goldstein DB, FALS Sequencing Consortium (2015). Exome sequencing in amyotrophic lateral sclerosis identifies risk genes and pathways. Science.

[26] Perkel JM (2017). Single-cell sequencing made simple. Nature.

