# Supplementary material for: Universal probe-based intermediate primer-triggered qPCR (UPIP-qPCR) for SNP genotyping
Source: BMC Genomics. 2021 Nov 24;22:850. doi: 10.1186/s12864-021-08148-2 (PMC8611915; doi:10.1186/s12864-021-08148-2)
Supplement: Supplementary file 1 — Additional file 1. Detailed Materials and Methods for UPIP-qPCR. [file 12864_2021_8148_MOESM1_ESM.docx]

## Detailed Materials and Methods for UPIP-qPCR

### 1. PCR and Sanger Sequencing

The nucleotide sequence containing specific SNPs were acquired from the dbSNP database of National Center for Biotechnology Information (NCBI, Bethesda, MD, USA) (https://www.ncbi.nlm.nih.gov/snp/). The primers flanking the SNP site (Fla-primers) and sequencing primers (Seq-primers) were designed using Primer Premier version 5 software. Sequences of Fla-primers and Seq-primers were listed in Table S1. Human genomic DNAs were extracted from whole blood samples via Rapid Blood Genomic DNA Isolation Kit according to the manufacturer's instructions (Sangon Biotech, Shanghai, China). Polymerase chain reactions (PCR) were performed to amplify 151–921 bp products containing targeted SNPs. For each SNP, the total PCR volume was 40 μL, containing 1× EmeraldAmp PCR Master Mix (TaKaRa, Shiga, Japan), genomic DNA (80 ng) and Fla-primers-F & R (500 nM/each). The PCR was programmed as follows: initial denaturation at 95 °C for 5 min, followed by 35 cycles of denaturation at 95 °C for 25 s, annealing at 62 °C for 30 s, elongation at 72 °C for 1 min, and final elongation at 72 °C for 5 min. PCR products were purified by gel extraction and used as templates in the dideoxy chain-termination PCR system using BigDye Terminator v3.1 Kit (Applied Biosystems, Foster City, CA, USA), the nucleotide sequences of the PCR products containing specific SNPs were obtained by the 3730xl DNA analyzer (Applied Biosystems).

### 2. UPIP-qPCR

UPIP-qPCR consisted of two separated reactions. The first stage (stage I) of UPIP-qPCR was a general PCR. For each SNP, the total stage I PCR volume was 20 μL, containing genomic DNA (20 ng), upstream site-specific primers and downstream specific primer (100 nM/each), ddH_2_O, dNTP, PCR buffer and hot-start DNA polymerase according the manufacturer's instructions (TaKaRa Taq™ Hot Start Version). In this stage, the reaction conditions were: initial denaturation at 95 °C for 3 min, followed by 10–22 cycles of denaturation at 98 °C for 5 s, annealing at 67 °C for 25 s, elongation at 72 °C for 20 s, and final elongation at 72 °C for 1 min. PCR products were diluted 10 times with ddH_2_O and used as templates in the second stage reaction. The stage I PCR was carried out using the T100^TM^ Thermal Cycler PCR machine (Bio-Rad, Hercules, CA, USA).

The second stage (stage II) of UPIP-qPCR was a qPCR. For each SNP, the total stage II PCR volume was 20 μL, containing template DNA (2 μL), intermediate primer (500 nM), upstream universal primer (500 nM) and universal probes (300 nM), and ddH_2_O, dNTP, PCR buffer and hot-start DNA polymerase according the manufacturer's instructions (TaKaRa Taq™ Hot Start Version). In this stage, the reaction conditions were: initial denaturation at 95 °C for 3 min, followed by 35–40 cycles of denaturation at 98 °C for 5 s, annealing at 49 °C for 25 s, elongation at 72 °C for 1 s and fluorescent signals were obtained via plate reading. The stage II qPCR was carried out on the CFX96 Touch qPCR machine (Bio-Rad). Sequences of primers for UPIP-qPCR were listed in Table S2. Sequences of FAM- and HEX-labeled universal probes and universal primer were listed in Table S3.

### 3. Optimization of UPIP-qPCR

In the optimization process of UPIP-qPCR, the upstream site-specific primers and downstream specific primer concentration in the stage I reaction system were set up in three gradients of 100 nM, 200 nM and 500 nM, stage I PCR reactions were set up in four different cycles of 10, 14, 18 and 22, and the PCR products were set up to four different dilutions of 0, 5, 10 and 20 times prior to use as templates in stage II reactions. The categories of reagent components in the stage I reaction system were not changed and is described in "2. UPIP-qPCR" of the methods. There were no changes in the primer and probe concentrations, reagent components, and reaction conditions in this optimization process, as described in "2. UPIP-qPCR".

### 4. Range Setting of Intermediate Primer’s Position

In the second stage of UPIP-qPCR, under the fixed amplification condition of extension at 72 ℃ for 1 s, six intermediate primers at different positions were set for the ALDH2 rs671 site, which were at a distance of 0, 30, 60, 90, 120, 150 bases from the 3′-end of intermediate primers to the SNP site. This was done to detect the effects due to the position of the intermediate primers on the generation of amplification curves and typical signals. The primer sequences are listed in Table S4. In this experiment, only the intermediate primers added in the stage II reaction system were different, and there were no changes in the other factors such as the reaction volume, reagent composition and reaction conditions, as described in "2. UPIP-qPCR" of the methods.

### 5. Sensitivity Analysis

The genomic DNA samples of the three genotypes of ALDH2 rs671 were used for UPIP-qPCR sensitivity analysis, and ddH_2_O was used NTC. In the first stage of UPIP-qPCR, the concentration gradients of genomic DNA were 100 ng, 10 ng, 1 ng, 0.1 ng, 0.01 ng, 0.003 ng and 0.001 ng per 10 μL reaction system. In the first stage reaction system, the primer concentration was 100 nM/each and there were 18 amplification cycles. Other reaction parameters and conditions were identical to the stage I reactions described in "2. UPIP-qPCR" of the methods. There were 40 amplification cycles in the second stage and the other reaction parameters and conditions were identical to the stage II reactions described in "2. UPIP-qPCR". TaqMan probe-qPCR was set as control method for the sensitivity analysis, the concentration gradients of genomic DNA were the same as UPIP-qPCR. The total volume of TaqMan probe-qPCR was 10μL, containing forward and reverse primers (500nM/each), probes (400nM/each), genomic DNA (1μL) and ddH_2_O, dNTP, PCR buffer and hot-start DNA polymerase according the manufacturer's instructions (TaKaRa Taq™ Hot Start Version). The TaqMan probe-qPCR reaction conditions were: initial denaturation at 95 °C for 3 min, followed by 40 cycles of denaturizing at 98 °C for 5 s, annealing at 62 °C for 25 s, elongating at 72 °C for 30 s and followed by plate reading. Sequences of primers and probes for TaqMan probe-qPCR were listed in Table S5.

### 6. Analysis of Call Rate and Accuracy for UPIP-qPCR

UPIP-qPCR was used to detect the genotypes of 224 human genomic DNA samples at rs671 (G > A), rs1057910 (A > C), rs9923231 (C > T) loci, , rs1801131, rs1801133, and rs1801394. The reaction system and detailed thermal cycle parameters can be found in "2. UPIP-qPCR" of the methods. In each experiment, DNA samples of three known genotypes with specific SNP loci were used as controls, while ddH_2_O was used as NTC. Reactions for each DNA sample of each SNP were repeated for three tubes, and only two or more tubes had the same fluorescence signals, then the result would be considered effective. The number of effective results with the total number of samples were compared to obtain the call rate of the UPIP-qPCR method, and the genotyping results were compared with the Sanger sequencing results of same samples to obtain the accuracy rate of UPIP-qPCR method. The UPIP-qPCR genotyping primers and Sanger sequencing primers for rs671, rs1057910, rs9923231, rs1801131, rs1801133, and rs1801394 are shown in Table S2 and Table S1, respectively. TaqMan probe-qPCR and KASP were set as control methods for the analysis of call rate and accuracy. The concentration of genomic DNA was 10ng/μL in TaqMan and KASP system. The reagent components and reaction conditions of TaqMan-qPCR were the same as that described in "5. Sensitivity analysis". Sequences of primers and probes for TaqMan probe-qPCR were listed in Table S5. The reagent components and reaction conditions of KASP were configured according to the manufacturer's instructions (KASP-TF V4.0 2X Master Mix). Sequences of primers for KASP were listed in Table S6.

### 7. Wide Applicability Analysis of UPIP-qPCR in SNP Genotyping

Based on UPIP-qPCR, we designed primers (Table S2) and genotyped 13 different SNPs, including rs10234411 (A > T), rs4961 (G > T), rs1801253 (G > C), rs1801131 (A > C), rs1801133 (C > T), rs394 (A > G), rs1045642 (T > C), rs3918290 (G > A), rs55886062 (A > C), rs1695 (A > G), rs25487 (A > G), rs35305379 (TTTA > TTTTA) and rs34481414 (ACTACAAT > ACAAT). These SNPs covered all SNP mutation types. The system volumes of the first and second stage UPIP-qPCR reactions were all 10 μL, and the cycle numbers of the first and second stage reactions were 10 and 40, respectively. For the reagent composition of the system, please refer to "2. UP- qPCR" of the methods. The reaction conditions of stage I were: initial denaturation at 95 °C for 3 min, followed by 10 cycles of denaturation at 95 °C for 20 s, annealing at 67 °C for 30 s (touchdown -0.4 °C/cycle), elongation at 72 °C for 30 s, and followed by 10 cycles of denaturation at 95 °C for 20 s, annealing at 63 °C for 30 s, elongation at 72 °C for 30 s, and final elongation at 72 °C for 2 min. Please refer to "2. UPIP-qPCR" for the reaction conditions of stage II.

### 8. Preparation of DNA Standards Containing Specific SNPs

Positive control samples were artificially constructed for all genotypes of all SNPs involved in this study. SNP loci were designed in the primers of positive control. As an internal reference, primers of human genome GAPDH was also designed to construct the positive control, however, the product was only wild-type. Primer sequences are shown in Table S8. For each genotype of an SNP, the total PCR volume was 50 μL, containing 1× EmeraldAmp PCR Master Mix (TaKaRa), genomic DNA (100 ng) and Control-wtF and/or Control-mutF & Control-R (500 nM /each). The PCR was programmed as follows: initial denaturation at 95 °C for 5 min, followed by 36 cycles of denaturation at 95 °C for 25 s, annealing at 65 °C for 30 s, elongation at 72 °C for 1 min, and final elongation at 72 °C for 5 min. PCR products were purified by gel extraction and re-dissolved in 50 μL ddH_2_O. DNA concentration was determined by Nanodrop 2000 (Thermo Fisher Scientific, USA) and diluted to 0.1 pg/μL as a working solution stored at 4 ℃. The equal volume mixture of wild-type positive control working solution of each SNP and the wild-type products of GAPDH formed the wild-type positive control substance of the multiplex PCR. Similarly, the mutant and heterozygous positive control substance of multiplex PCR were also obtained by this method.

### 9. Multiplex UPIP-qPCR

In the stage I reactions, 16 types of SNPs genotyping primers and GAPDH primers were mixed in proportion to form 17 multi-primer working solutions. Although the concentration of upstream and downstream primers of a specific SNP was equal, the final concentration in the reaction system of each SNP were not equal. rs671, rs1057910, rs9923231, rs10234411, rs4961, rs1801131, rs1801133, rs394, rs3918290, rs55886062 and rs1695 primers had a final concentration of 12.96 nM; rs1045642, rs25487, rs35305379 and rs34481414 primers had a final concentration of 38.87 nM; and rs1801253 primer had a final concentration of 77.74nM and the GAPDH primer had a final concentration of 4.28 nM. For effective amplification, the ratio of these four concentrations was 3:9:18:1. Seventeen multi-primer working solutions were prepared by mixing 2 μM original primers of each SNP according to the volume ratio of the above proportion. The total volume of multiplex PCR system was 20 μL, including 10 μL 2× buffer, 0.4 μL multiplex DNA polymerase (Vazyme, PM101), 2μL DNA (10 ng/μL), 7.6 μL of 17 multi-primer working solution. The reaction conditions of multiplex PCR were: initial denaturation at 95°C for 5 min, followed by 10 cycles of denaturation at 95 °C for 20 s, annealing at 67 °C for 30 s (touchdown -0.4 °C/cycle), elongation at 72 °C for 30 s, and followed by 10 cycles of denaturation at 95 °C for 20 s, annealing at 63 °C for 30 s, elongation at 72 °C for 30 s, and final elongation at 72 °C for 5 min. The first stage products were digested by exonuclease I (Exo I, Takara) to remove redundant primers prior to use as templates for the second stage reactions. The Exo I reaction system consisted of 2 μL products of stage I, 2 μL 10× buffer, 1 μL Exo I (5 U/μL), 15 μL ddH_2_O. The Exo I reaction conditions were 37 ℃ for 30 min and 85 ℃ for 15 min. The second stage were single reactions, please refer to "2. UPIP-qPCR" of methods for the detailed reaction conditions. The template used in the first stage reactions was human genome DNA sample No. 1, ddH_2_O was used as NTC. Correctness was defined by comparing the experimental results with that of the Sanger sequencing.

### 10. DAN Microarray Assay

The microarrays integrated 20 types of probes with five duplicates, these probes included intermediate primers of 16 types of SNPs, complementary sequences of FAM and HEX single-labeled primers as positive reference, intermediate primers of GAPDH as internal reference, and amino modified 14-poly deoxythymine (NH_2_-dT_14_) as negative control probe. The NH_2_-dT_14_ was added to each 5′-terminal of all these primers prior to use. Probes were injected on an aldehyde modified glass slide with a concentration of 10 μM/each. See Table S9 for sequences of these probes. The microarrays were stored at 4 ℃ or directly used after fixation overnight at room temperature (Microarrays were constructed by Qingdao OE Biotech).

The preparation process of PCR products for hybridization was divided into three stages. The first stage was a multiplex PCR. Please see "Multiplex UPIP-qPCR" of methods for the detailed reaction system and parameters. A universal reverse connector, 5′-TGGGAGCTGAGGGCGA-3′, was added to the 5′-terminal of the downstream specific primer of each SNP, and the primer with the same sequence was called the universal reverse primer. In the first stage, normal human genome DNA (No. 1 & No. 2) were used as the template DNA of the experimental group, and the template DNA of the control groups was divided into three types, namely, the mixture of wild type, heterozygous and mutant positive control DNA of 16 SNP sites, and the wild type positive control DNA of GAPDH, respectively. ddH_2_O was used as the blank control template.

The second stage was a product treatment process, i.e. using Exo I to digest the products of the first stage which would be used as templates in the third stage reactions. Please refer to "9. Multiplex UPIP-qPCR" of methods for the detailed reaction system and parameters of this digestion process. The third stage was the fluorescence labeling PCR reaction. FAM and HEX single-labeled primers and universal reverse primer (Table S3) were combined to amplify the templates processed by Exo I to obtain sufficient DNA fragments for microarray hybridization. The total stage III PCR volume was 30 μL, containing 4 μL template DNA, 667 nM FAM single-labeled primer, 667 nM HEX single-labeled primer, 667 nM universal reverse primer and ddH_2_O, dNTP, PCR buffer and hot-start DNA polymerase according the manufacturer's instructions (TaKaRa Taq™ Hot Start Version). In this stage, the reaction conditions were: initial denaturation at 95 °C for 3 min, followed by 40 cycles of denaturation at 95 °C for 25 s, annealing at 52 °C for 30 s, elongation at 72 °C for 20 s.

The microarrays were placed in a pre-hybridizing solution (25% formamide, 5× SSC, 0.1% SDS, 0.5% BSA) at 42 ℃ for 5 min in water bath. Products of the third stage were denatured at 95 ℃ for 5 min, cooled on ice for 2 min, then mixed with equal volume (30 μL) hybridizing buffer (50% formamide, 10× SSC, 0.2% SDS) to form hybridizing solution. 20 μL of hybridizing solution was aliquoted into the microarrays, covered with coverslips, and incubated at 40 ℃ for 16 h–20 h. Following hybridization, microarrays were transferred into cleaning solution A (0.1× SSC, 0.1% SDS) for 5 min at room temperature (18–25°C), then transferred into cleaning solution B (0.1× SSC) for 5 min, and dehydrated with 100% alcohol for 10 s. After drying at room temperature (18–25°C), the images of FAM and HEX signals were captured using a confocal microscope (Leica TCS SP8, Leica, Wetzlar, Germany). By merging the FAM and HEX signal images of the same field, the genotyping results of the samples could be obtained. Specifically, dots with only FAM signals (green) were homozygous wild type, those with only HEX signals (red) were homozygous mutant, and those with both signals (yellow) were heterozygous.

## The characteristics of the primers and probes involved in UPIP-qPCR

The upstream site-specific primer is divided into two parts: (1) the 5′-end, which is a universal connector composed of the same sequence of 14-base upstream universal primers and the reverse complementary sequence of 18-base universal probe, and (2) the 3′-end, which is the upstream specific primer sequence combining with the template DNA, and whose 3′-terminal base is designed according to the specific SNP genotype (Figure 1a). As most of the SNPs are dimorphic and only few are polymorphic, there are generally two upstream site-specific primers in a reaction system to distinguish the different base types.

The downstream specific primer combines to the template DNA to complete the first stage of the PCR reaction together with upstream site-specific primers, and provides the template for the second stage reaction (Figure 1a).

The universal primer is a fixed-sequence primer with –14 bases, and a sequence identical to the 5′-terminal segment in the upstream specific primer. The universal primer acts as the upstream primer, and cooperates with the intermediate primer to complete the reaction of the second stage of UPIP-qPCR (Figure 1a).

The intermediate primer, likes the downstream nested primer, is complementary to the template between the upstream site-specific primer and the downstream specific primer; the 5′-3′ direction is the same as that of the downstream specific primer. The primer plays a role in targeting the correct template and triggering specific amplification and signal release in the second stage reaction. Intermediate primers are fundamental in the specificity of this technology (Figure 1a).

This universal probe, like the TaqMan probe, is designed based on the principle of FRET and is composed of a fluorophore at one end and a quencher at the other with its sequence reverse complementary to the middle part of the upstream site-specific primer. In the second stage, DNA polymerases containing 5′-3′ exonuclease activity initiate the hydrolysis of universal probes and release fluorescence signals (Figure 1a). In this reaction system, two types of universal probes with different sequences are designed and tagged with different fluorescent labels to differentiate between two alleles signals of an SNP. In the reaction system of this study, to facilitate the genotyping of SNPs, FAM signals were set to represent wild-type alleles and HEX signals were set to represent mutant alleles.
