# Supplementary material for: Universal probe-based intermediate primer-triggered qPCR (UPIP-qPCR) for SNP genotyping
Source: BMC Genomics. 2021 Nov 24;22:850. doi: 10.1186/s12864-021-08148-2 (PMC8611915; doi:10.1186/s12864-021-08148-2)
Supplement: Supplementary file 2 — Additional file 2. Additional results of UPIP-qPCR. [file 12864_2021_8148_MOESM2_ESM.docx]

**Additional results of UPIP-qPCR**

## 1. The characteristics of the primers and probes involved in UPIP-qPCR

The upstream site-specific primer is divided into two parts: (1) the 5′-end, which is a universal connector composed of the same sequence of 14-base upstream universal primers and the reverse complementary sequence of 18-base universal probe, and (2) the 3′-end, which is the upstream specific primer sequence combining with the template DNA, and whose 3′-terminal base is designed according to the specific SNP genotype (Figure 1A). As most of the SNPs are dimorphic and only few are polymorphic, there are generally two upstream site-specific primers in a reaction system to distinguish the different base types.

The downstream specific primer combines to the template DNA to complete the first stage of the PCR reaction together with upstream site-specific primers, and provides the template for the second stage reaction (Figure 1a).

The universal primer is a fixed-sequence primer with –14 bases, and a sequence identical to the 5′-terminal segment in the upstream specific primer. The universal primer acts as the upstream primer, and cooperates with the intermediate primer to complete the reaction of the second stage of UPIP-qPCR (Figure 1a).

The intermediate primer, likes the downstream nested primer, is complementary to the template between the upstream site-specific primer and the downstream specific primer; the 5′-3′ direction is the same as that of the downstream specific primer. The primer plays a role in targeting the correct template and triggering specific amplification and signal release in the second stage reaction. Intermediate primers are fundamental in the specificity of this technology (Figure 1a).

This universal probe, like the TaqMan probe, is designed based on the principle of FRET and is composed of a fluorophore at one end and a quencher at the other with its sequence reverse complementary to the middle part of the upstream site-specific primer. In the second stage, DNA polymerases containing 5′-3′ exonuclease activity initiate the hydrolysis of universal probes and release fluorescence signals (Figure 1a). In this reaction system, two types of universal probes with different sequences are designed and tagged with different fluorescent labels to differentiate between two alleles signals of an SNP. In the reaction system of this study, to facilitate the genotyping of SNPs, FAM signals were set to represent wild-type alleles and HEX signals were set to represent mutant alleles.

**2.** **Optimization of UPIP-qPCR**

Based on the feasibility of UPIP-qPCR, we optimized the primer concentration, amplification cycle number and template dilution ratio of the first stage reaction, to ensure the stability and reliability of UPIP-qPCR technology, and to reduce the detection cost and duration as much as possible. The final concentration of PCR primers is generally 500nM/each. In the optimization process, we set up three gradients of 100 nM, 200 nM and 500 nM for the upstream and downstream specific primers, set at 10-, 14-, 18- and 22-different cycles for the PCR reaction, and set to 0-, 5-, 10- and 20-times dilutions for the PCR products, to identify the effects of these experimental factors on the curves and results of the second stage reaction. The results showed that only the products of the 100 nM/each primer concentration and 10 cycles amplification could be directly used as templates for the second stage without dilution (1 μL template/10 μL system). In all other conditions, the products should be diluted before used as templates (Figure S2A–S2D).

To further shorten the detection period, the duration of the elongation step at 72 ℃ in the second stage was set to 1s, and signal acquisition was directly conducted. A suitable distance, theoretically, the closer the better, between the 3′-end of the intermediate primer and the SNP site was required. Based on the genotyping experiment of *ALDH2* rs671 (G > A), we designed five intermediate primers with a base distance of 0, 30, 60, 90 and 120 from their 3′-terminal to the SNP site, and observed the effect of the different intermediate primers on the amplification efficiency and genotyping results, based on which we formulated the optimal design principle of intermediate primers. The results showed that the intermediate primers with the base distance of 0, 30 and 60 had good amplification efficiency (Figure S3A) and acquired accurate genotyping results for all three different genotypic DNA samples (Figure S3B); however, the RFU value of reactions with a base distance of 0, and 30 was significantly higher than that of 60 bases (*p* < 0.01) (Figure S3C– S3E). Thus, to ensure the difference in the RFU values, the intermediate primers should be designed within a distance of 30 bases from its 3′-terminal to the SNP site.

**3. Multiplex PCR can be adopted in stage I reactions**

To reduce the complexity of the reaction and the consumption of template DNA, we mixed the primers involved in the first stage reaction of 16 SNPs in one tube to perform a multiplex PCR. The product was diluted ten times and digested by Exo I enzyme before used as the template of the second stage reaction. The results showed that specific amplification signals and typical amplification curves were obtained in the second stage reaction from the DNA sample (DNA No. 1) (Figure S7A), NTC had no false positive signals (Figure S7B), and the genotyping results were consistent with that of Sanger sequencing (Table S10).
