# Supplementary figures and images for: Universal probe-based intermediate primer-triggered qPCR (UPIP-qPCR) for SNP genotyping
Source: BMC Genomics. 2021 Nov 24;22:850. doi: 10.1186/s12864-021-08148-2 (PMC8611915; doi:10.1186/s12864-021-08148-2)

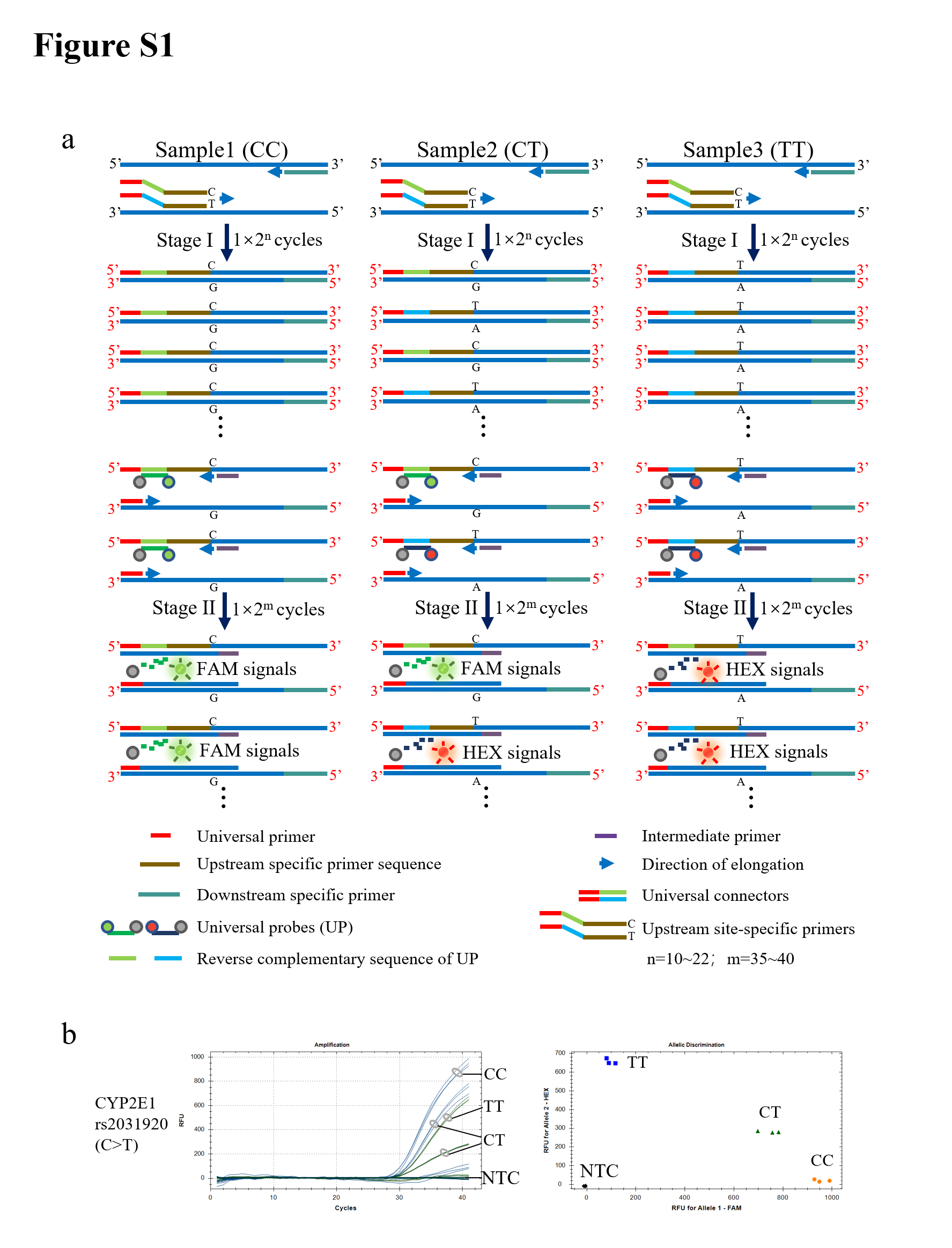

Supplement: Supplementary file 4 — Additional file 4. Supplementary figure legends. [file 12864_2021_8148_MOESM4_ESM.zip › updated additional files/FigS1_ESM.tif]

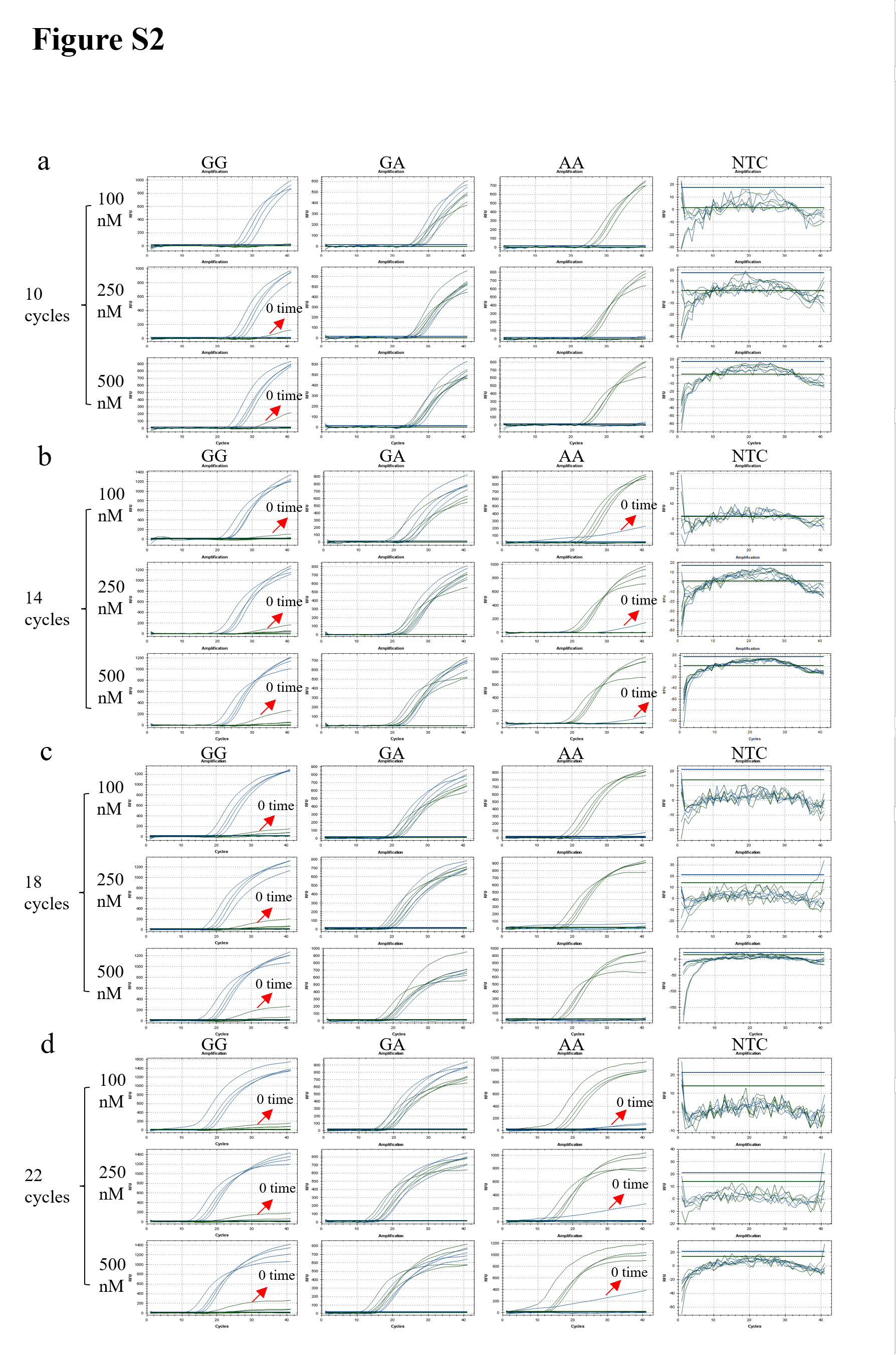

Supplement: Supplementary file 4 — Additional file 4. Supplementary figure legends. [file 12864_2021_8148_MOESM4_ESM.zip › updated additional files/FigS2_ESM.tif]

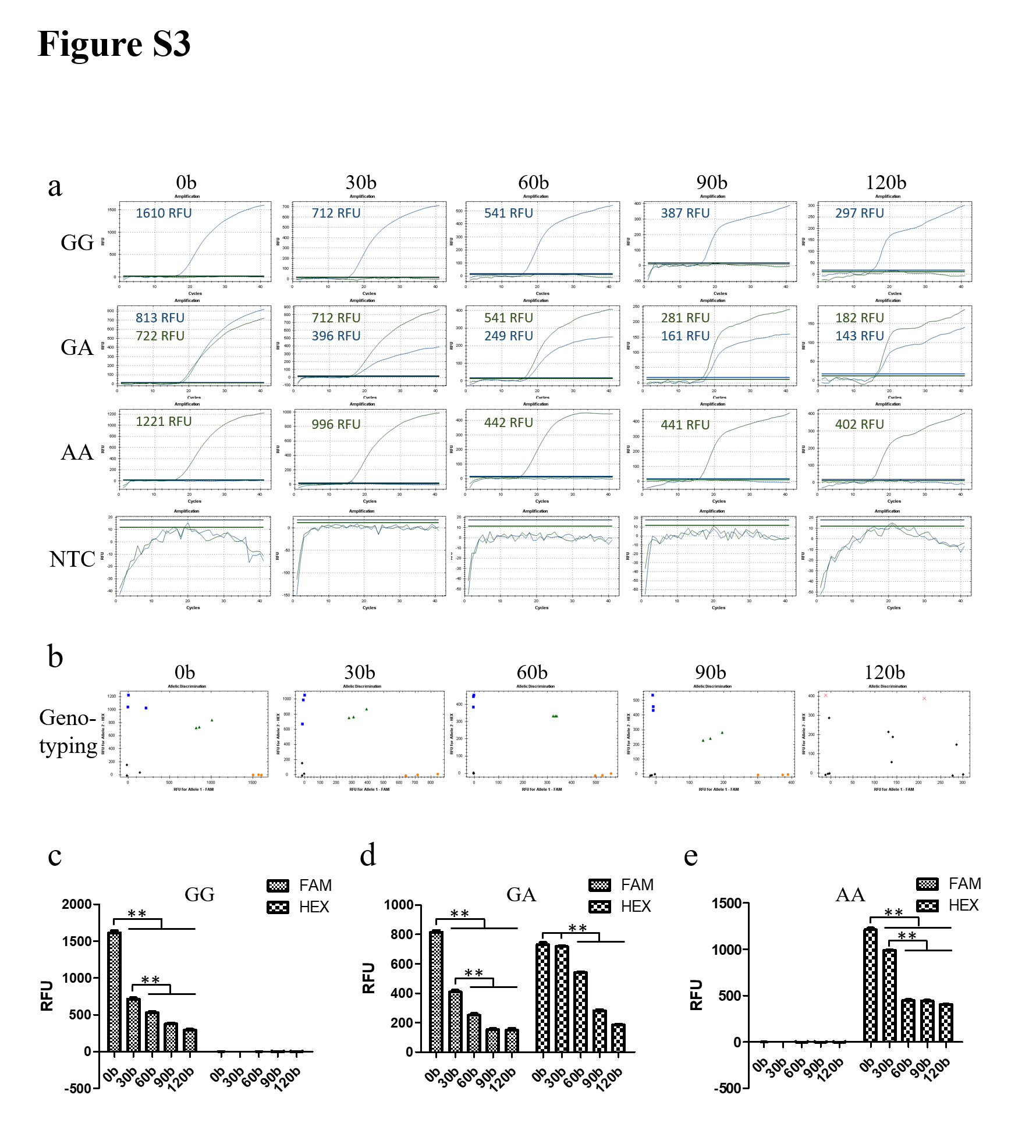

Supplement: Supplementary file 4 — Additional file 4. Supplementary figure legends. [file 12864_2021_8148_MOESM4_ESM.zip › updated additional files/FigS3_ESM.tif]

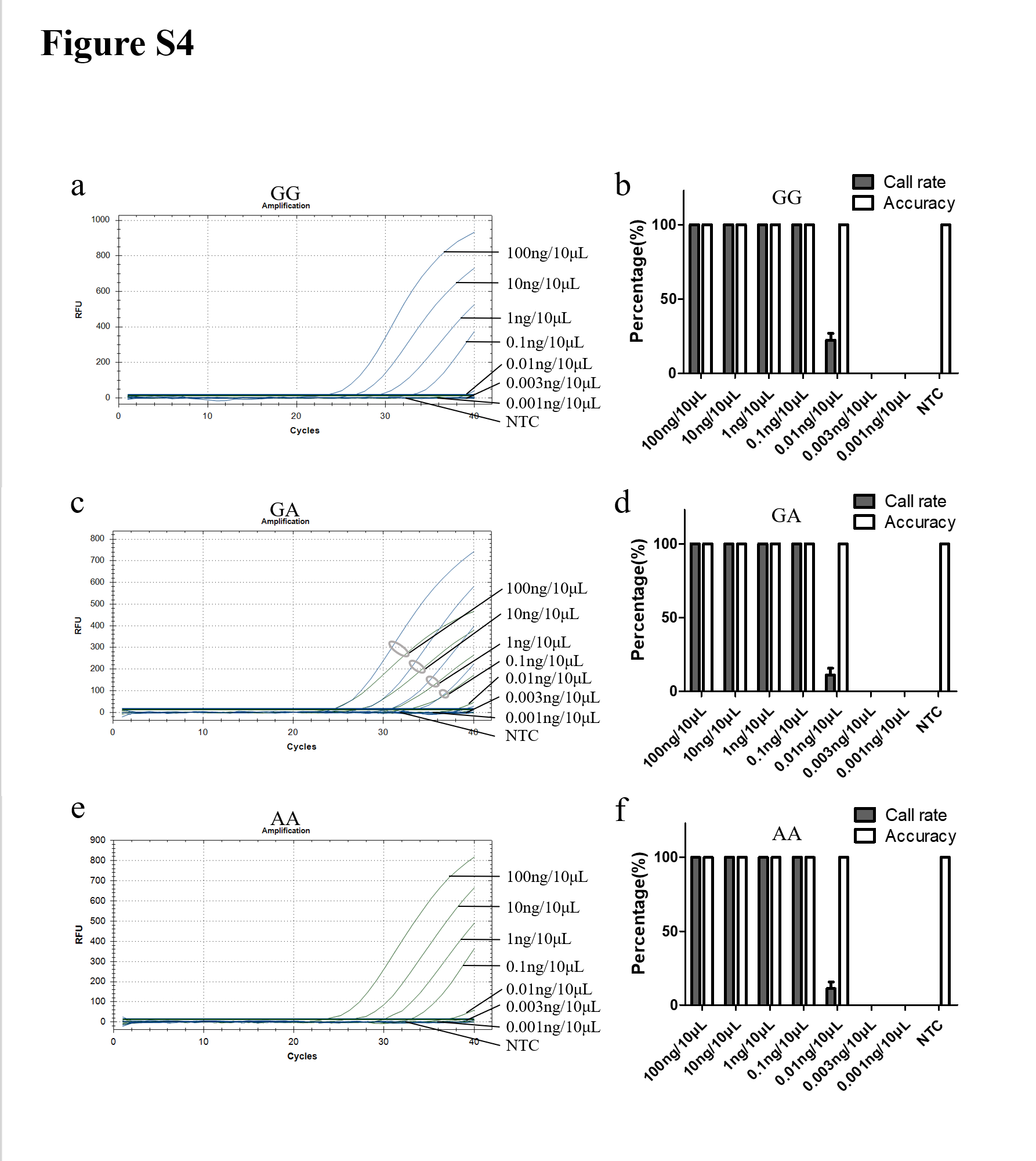

Supplement: Supplementary file 4 — Additional file 4. Supplementary figure legends. [file 12864_2021_8148_MOESM4_ESM.zip › updated additional files/FigS4_ESM.tif]

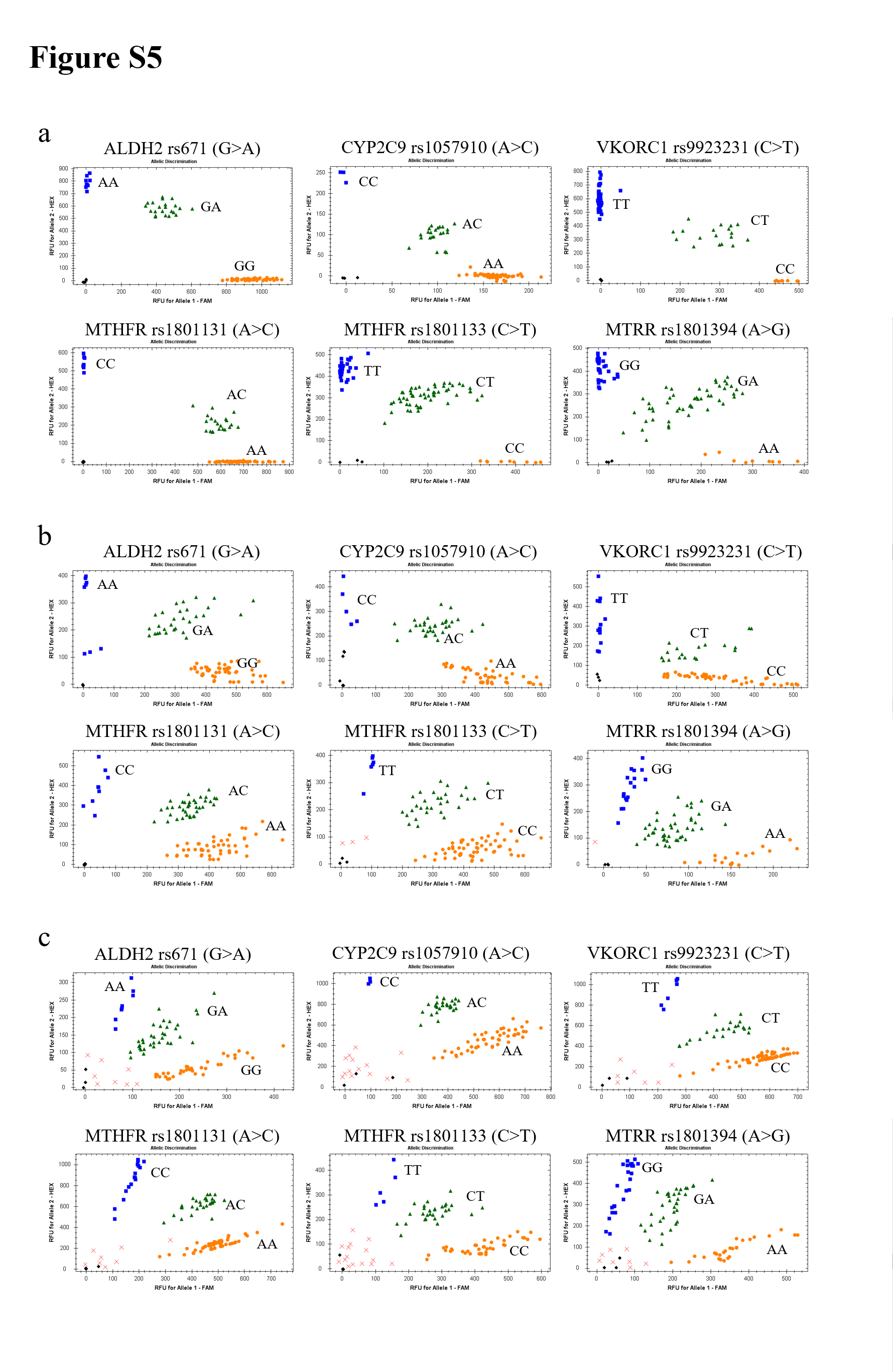

Supplement: Supplementary file 4 — Additional file 4. Supplementary figure legends. [file 12864_2021_8148_MOESM4_ESM.zip › updated additional files/FigS5_ESM.tif]

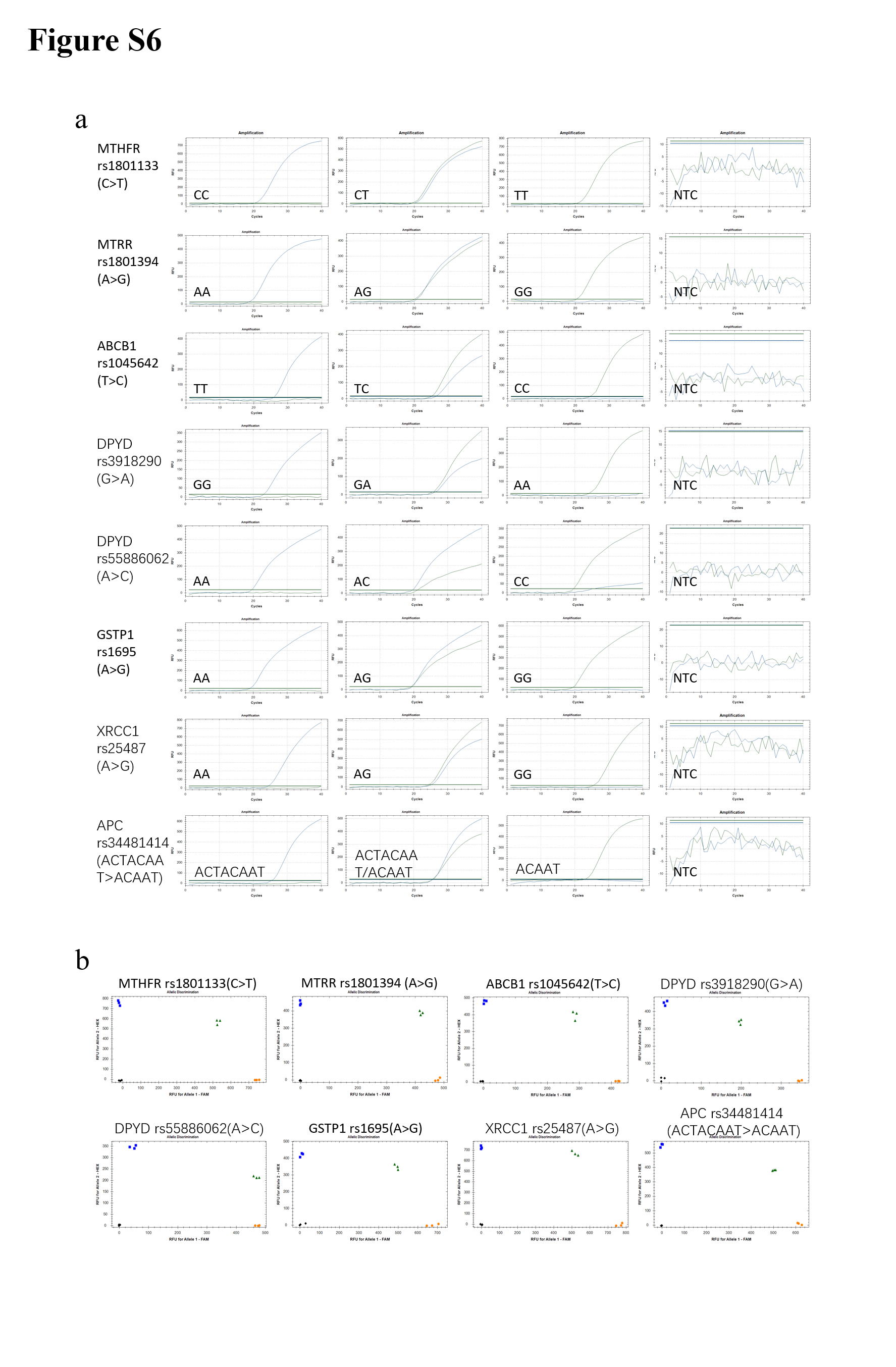

Supplement: Supplementary file 4 — Additional file 4. Supplementary figure legends. [file 12864_2021_8148_MOESM4_ESM.zip › updated additional files/FigS6_ESM.tif]

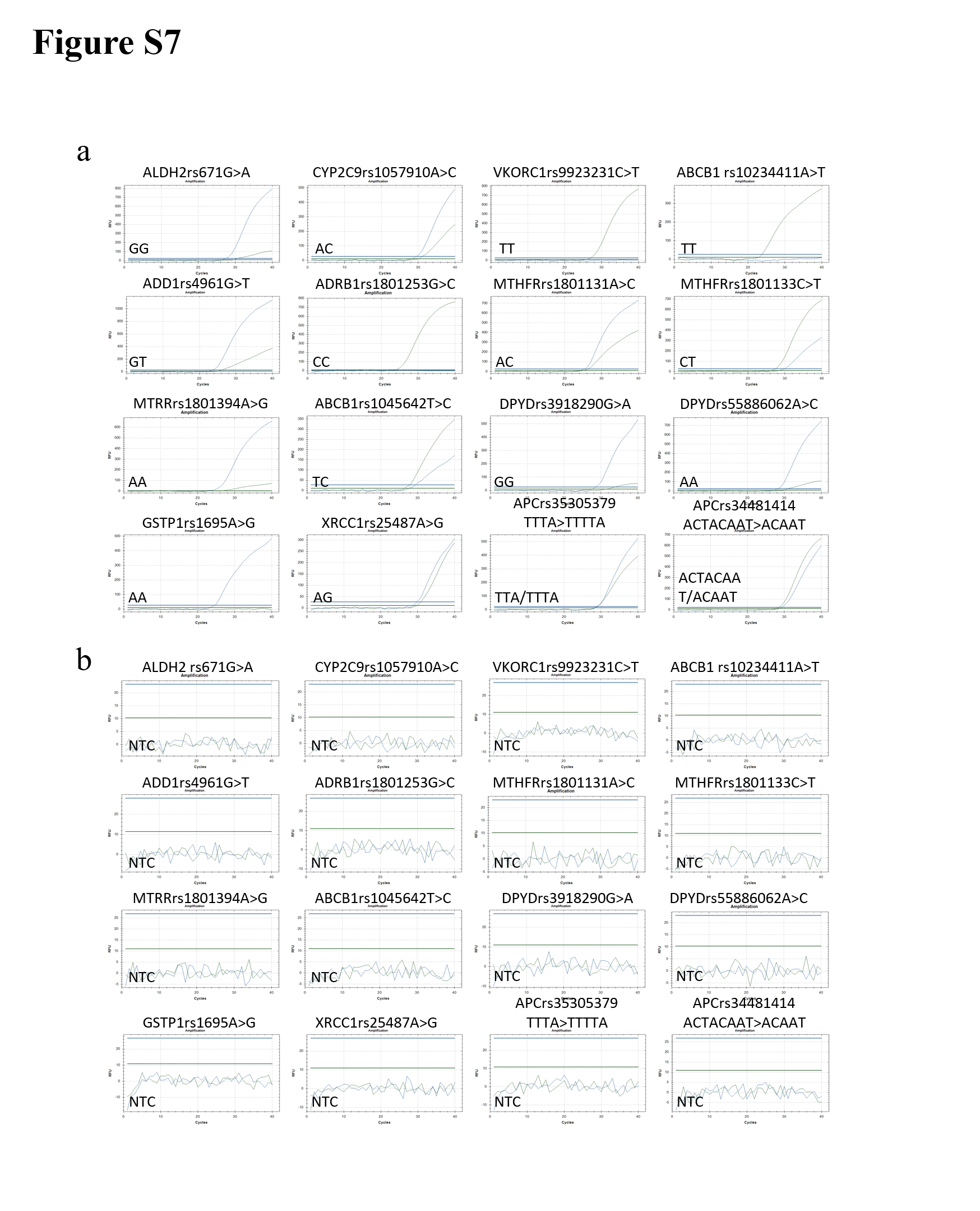

Supplement: Supplementary file 4 — Additional file 4. Supplementary figure legends. [file 12864_2021_8148_MOESM4_ESM.zip › updated additional files/FigS7_ESM.tif]
