# Supplementary material for: Universal probe-based intermediate primer-triggered qPCR (UPIP-qPCR) for SNP genotyping
Source: BMC Genomics. 2021 Nov 24;22:850. doi: 10.1186/s12864-021-08148-2 (PMC8611915; doi:10.1186/s12864-021-08148-2)
Supplement: Supplementary file 5 — Additional file 5. Supplementary Tables. [file 12864_2021_8148_MOESM5_ESM.docx]

| **Table S1** Primers for Sanger Sequencing | | | | | |
| --- | --- | --- | --- | --- | --- |
| Gene | Rs. No. | Variation | Primer type | Primer sequence (5'-3') | Product (bp) |
| ALDH2 | rs671 | G>A | Fla-primer-F | CAGATGTGGAGGTTGCAACGAG | 378 |
|  |  |  | Fla-primer-R | TCCGAGCCACCAGCAGAC |  |
|  |  |  | Seq-primer | TCAACTGCTATGATGTGTTTGGAGC |  |
| CYP2E1 | rs2031920 | C>T | Fla-primer-F | TTCTATGAAGGTAGTCCATAGGTATTT | 160 |
|  |  |  | Fla-primer-R | AGCAGCCCCCAGTAGTACCT |  |
|  |  |  | Seq-primer | AAACCAGAGGGAAGCAAAGGC |  |
| CYP2C9 | rs1057910 | A>C | Fla-primer-F | TCCAGGAAGAGATTGAACGTGTG | 200 |
|  |  |  | Fla-primer-R | TAGGAGAAACAAACTTACCTTGGGA |  |
|  |  |  | Seq-primer | TGAACGTGTGATTGGCAGAAAC |  |
| VKORC1 | rs9923231 | C>T | Fla-primer-F | CGGTGCCATCTCGGCTCA | 231 |
|  |  |  | Fla-primer-R | GGAAGTCAAGCAAGAGAAGACCTG |  |
|  |  |  | Seq-primer | GTGATCCACCCACCTCGGC |  |
| ABCB1 | rs10234411 | A>T | Fla-primer-F | TGCAATCTCAGCTCTGCAACCT | 399 |
|  |  |  | Fla-primer-R | CCCCTCTTGAACCTTATCCATCTT |  |
|  |  |  | Seq-primer | CACCTCCTGAGTTCAAGTAATTCTC |  |
| ADD1 | rs4961 | G>T | Fla-primer-F | TAGTGTCACAGGTTACTCCTTTGCT | 288 |
|  |  |  | Fla-primer-R | TGAACAGTAAGGTAGGCACAGACCA |  |
|  |  |  | Seq-primer | GTGACGGTGATTCGGGCA |  |
| ADRB1 | rs1801253 | G>C | Fla-primer-F | GCCTCTTCGTCTTCTTCAACTGG | 151 |
|  |  |  | Fla-primer-R | TCTCCGTGGGTCGCGTGGC |  |
|  |  |  | Seq-primer | TGGGCTACGCCAACTCGG |  |
| MTHFR | rs1801131 | A>C | Fla-primer-F | TGCTCCCCTGCTTCCGGC | 921 |
|  |  |  | Fla-primer-R | GGTTCACCCGCAGCAGCT |  |
|  |  |  | Seq-primer | TCTACCTGAAGAGCAAGTCCCC |  |
| MTHFR | rs1801133 | C>T | Fla-primer-F | TGGAAGGTGCAAGATCAGAGCC | 427 |
|  |  |  | Fla-primer-R | GGGAAGAACTCAGCGAACTCAGC |  |
|  |  |  | Seq-primer | CTCCTGACTGTCATCCCTATTGG |  |
| MTRR | rs1801394 | A>G | Fla-primer-F | TTCAAGCCCAAGTAGTTTCGAGC | 420 |
|  |  |  | Fla-primer-R | GGCTCTAACCTTATCGGATTCACTAATA |  |
|  |  |  | Seq-primer | GTTTCACTGTTACATGCCTTGAAGT |  |
| ABCB1 | rs1045642 | T>C | Fla-primer-F | TTGATCTGTGAACTCTTGTTTTCAGC | 315 |
|  |  |  | Fla-primer-R | GGAGACCAGCCCCTTATAAATCAA |  |
|  |  |  | Seq-primer | AGCGACTGAATGTTCAGTGGC |  |
| DPYD | rs3918290 | G>A | Fla-primer-F | CAAATGTTTCCCCCAGAATCATC | 288 |
|  |  |  | Fla-primer-R | ATCAGCAAAGCAACTGGCAGATT |  |
|  |  |  | Seq-primer | CCCCAGAATCATCCGGGGA |  |
| DPYD | rs55886062 | A>C | Fla-primer-F | AATATATGCCTGCCCCTTCTTCC | 383 |
|  |  |  | Fla-primer-R | GCCAAGCCTGAACTACCCCTC |  |
|  |  |  | Seq-primer | GGGACAGAAAGGAAGGAAAGAAACT |  |
| GSTP1 | rs1695 | A>G | Fla-primer-F | TCCTTCCACGCACATCCTCTT | 327 |
|  |  |  | Fla-primer-R | CCAAGCCACCTGAGGGGTAAG |  |
|  |  |  | Seq-primer | CATCCCCAGTGACTGTGTGTTGA |  |
| XRCC1 | rs25487 | A>G | Fla-primer-F | CCTCAGATCACACCTAACTGGCATC | 305 |
|  |  |  | Fla-primer-R | GCCCCGCTCCTCTCAGTAGTCT |  |
|  |  |  | Seq-primer | ACCCCCAAGTACAGCCAGGTC |  |
| APC | rs35305379 | TTTA>TTTTA | Fla-primer-F | CACTCTCCTCATTAAACAATGACTGA | 388 |
|  |  |  | Fla-primer-R | AAACTAGAGAAATAAAGTGAATAAGTGGTGA |  |
|  |  |  | Seq-primer | GCAAAGGTACATCTACAGATGGAA |  |
| APC | rs34481414 | ACTACAAT>ACAAT | Fla-primer-F | AGCATTCAGCTAAATTATTAGTTATAAAGAT | 326 |
|  |  |  | Fla-primer-R | AAAACACTGCCAAATGTTGTCCA |  |
|  |  |  | Seq-primer | CTGTATCACTGTTGAATGGAGAGAA |  |
| Fla-primer and Seq-primer represent the primers flanking the SNP site and primers for sequencing reactions respectively. | | | | | |

| **Table S2** Primers for UPIP-qPCR | | | | | |
| --- | --- | --- | --- | --- | --- |
| Gene | Rs. No. | Variation | Primer type | Primer sequence (5'-3') | Product (bp) |
| ALDH2 | rs671 | G>A | UPq-WT-F | CTGTCCTCGGCACGCAGGGAAGGTGGTAGGTGCCCACACTCACAGTTTTCAATTC | 218 |
|  |  |  | UPq-Mut-F | CTGTCCTCGGCACGGTGATTTGGTGGGAGGAGCCCACACTCACAGTTTTCACTTT |  |
|  |  |  | UPq-R | CCTGGGAGTGTAACCCATAACC |  |
|  |  |  | Intermediate | GGCTGCAGGCATACACT |  |
| CYP2E1 | rs2031920 | C>T | UPq-WT-F | TTCTATGAAGGTAGTCCATAGGTATTT | 192 |
|  |  |  | UPq-Mut-F | CTGTCCTCGGCACGCAGGGAAGGTGGTAGGTGCATAAAGATTCATTGTTAATATAAAAGTAC |  |
|  |  |  | UPq-R | CTGTCCTCGGCACGGTGATTTGGTGGGAGGAGCATAAAGATTCATTGTTAATATAAAAGTAT |  |
|  |  |  | Intermediate | GAAGTTCTTAATTCATAGGTTGC |  |
| CYP2C9 | rs1057910 | A>C | UPq-WT-F | CTGTCCTCGGCACGCAGGGAAGGTGGTAGGTGAGTGCACGAGGTCCAGAGATACA | 151 |
|  |  |  | UPq-Mut-F | CTGTCCTCGGCACGGTGATTTGGTGGGAGGAGAGTGCACGAGGTCCAGTGATACC |  |
|  |  |  | UPq-R | TAGGAGAAACAAACTTACCTTGGGA |  |
|  |  |  | Intermediate | GTGGGGAGAAGGTCAA |  |
| VKORC1 | rs9923231 | C>T | UPq-WT-F | CTGTCCTCGGCACGCAGGGAAGGTGGTAGGTGAGCGTGAGCCACCGCTCCC | 248 |
|  |  |  | UPq-Mut-F | CTGTCCTCGGCACGGTGATTTGGTGGGAGGAGAGCGTGAGCCACCGAACCT |  |
|  |  |  | UPq-R | TGGTGACCATTATTCTGTCTACCACACT |  |
|  |  |  | Intermediate | GACCTGAAAAACAACCAT |  |
| ABCB1 | rs10234411 | A>T | UPq-WT-F | CTGTCCTCGGCACGCAGGGAAGGTGGTAGGTGAAGGCATAGTACCAGCTCCTCTTT | 237 |
|  |  |  | UPq-Mut-F | CTGTCCTCGGCACGGTGATTTGGTGGGAGGAGAAGGCATAGTACCAGCTCCTCTTA |  |
|  |  |  | UPq-R | TGCAATCTCAGCTCTGCAACCT |  |
|  |  |  | Intermediate | GTTTGTATTAAAACAACAATTAC |  |
| ADD1 | rs4961 | G>T | UPq-WT-F | CTGTCCTCGGCACGCAGGGAAGGTGGTAGGTGCGACGAAGCTTCCGAGGAAG | 203 |
|  |  |  | UPq-Mut-F | CTGTCCTCGGCACGGTGATTTGGTGGGAGGAGCGACGAAGCTTCCGACGAAT |  |
|  |  |  | UPq-R | TGAACAGTAAGGTAGGCACAGACCA |  |
|  |  |  | Intermediate | GGACTGCTTCCATTCT |  |
| ADRB1 | rs1801253 | G>C | UPq-WT-F | CTGTCCTCGGCACGCAGGGAAGGTGGTAGGTGCCGCAAGGCCTACCAGG | 104 |
|  |  |  | UPq-Mut-F | CTGTCCTCGGCACGGTGATTTGGTGGGAGGAGCCGCAAGGCCTTACAGC |  |
|  |  |  | UPq-R | TCTCCGTGGGTCGCGTGGC |  |
|  |  |  | Intermediate | GCGCAGCAGAGCAGT |  |
| MTHFR | rs1801131 | A>C | UPq-WT-F | CTGTCCTCGGCACGCAGGGAAGGTGGTAGGTGAAGGAGGAGCTGACCAGTGAAGA | 140 |
|  |  |  | UPq-Mut-F | CTGTCCTCGGCACGGTGATTTGGTGGGAGGAGTTGGAGGAGCTGACCAGTTAAGC |  |
|  |  |  | UPq-R | GGTCCCCACTCCAGCATCACT |  |
|  |  |  | Intermediate | GGTTCTCCCGAGAGGT |  |
| MTHFR | rs1801133 | C>T | UPq-WT-F | CTGTCCTCGGCACGCAGGGAAGGTGGTAGGTGTGAGAAGGTGTCTGCGGAAGC | 277 |
|  |  |  | UPq-Mut-F | CTGTCCTCGGCACGGTGATTTGGTGGGAGGAGTGAGAAGGTGTCTGCGGGTGT |  |
|  |  |  | UPq-R | GGGAAGAACTCAGCGAACTCAGC |  |
|  |  |  | Intermediate | AGCCTCAAAGAAAAGCT |  |
| MTRR | rs1801394 | A>G | UPq-WT-F | CTGTCCTCGGCACGCAGGGAAGGTGGTAGGTGCATGTACCACAGCTTGCTCTCAT | 156 |
|  |  |  | UPq-Mut-F | CTGTCCTCGGCACGGTGATTTGGTGGGAGGAGCATGTACCACAGCTTGCTAACAC |  |
|  |  |  | UPq-R | CCATTTTTCAGTTTCACTGTTACATGC |  |
|  |  |  | Intermediate | AGGCCATCGCAGAAGA |  |
| ABCB1 | rs1045642 | T>C | UPq-WT-F | CTGTCCTCGGCACGCAGGGAAGGTGGTAGGTGAGTGGTGTCACAGGAAGAGTTT | 192 |
|  |  |  | UPq-Mut-F | CTGTCCTCGGCACGGTGATTTGGTGGGAGGAGAGGTGGTGTCACAGGATGAGATC |  |
|  |  |  | UPq-R | GGAGACCAGCCCCTTATAAATCAA |  |
|  |  |  | Intermediate | TTGGCCTCCTTTGC |  |
| DPYD | rs3918290 | G>A | UPq-WT-F | CTGTCCTCGGCACGCAGGGAAGGTGGTAGGTGAAGGCTGACTTTCCAGATAACG | 186 |
|  |  |  | UPq-Mut-F | CTGTCCTCGGCACGGTGATTTGGTGGGAGGAGAAGGCTGACTTTCCAGACAACA |  |
|  |  |  | UPq-R | ATCAGCAAAGCAACTGGCAGATT |  |
|  |  |  | Intermediate | TTGTTTTAGATGTTAAATCACA |  |
| DPYD | rs55886062 | A>C | UPq-WT-F | CTGTCCTCGGCACGCAGGGAAGGTGGTAGGTGGCCACCAGCACATCAATGAT | 279 |
|  |  |  | UPq-Mut-F | CTGTCCTCGGCACGGTGATTTGGTGGGAGGAGGCCACCAGCACATCAATGAG |  |
|  |  |  | UPq-R | GGGACAGAAAGGAAGGAAAGAAACT |  |
|  |  |  | Intermediate | CTTCAAAAGCTCTTCGA |  |
| GSTP1 | rs1695 | A>G | UPq-WT-F | CTGTCCTCGGCACGCAGGGAAGGTGGTAGGTGGGACCTCCGCTGCAAATACA | 166 |
|  |  |  | UPq-Mut-F | CTGTCCTCGGCACGGTGATTTGGTGGGAGGAGGGACCTCCGCTGCATATACG |  |
|  |  |  | UPq-R | CCAAGCCACCTGAGGGGTAAG |  |
|  |  |  | Intermediate | TTGGTGTAGATGAGGGAGA |  |
| XRCC1 | rs25487 | A>G | UPq-WT-F | CTGTCCTCGGCACGCAGGGAAGGTGGTAGGTGAGCGTGTGAGGCCTTTCCTCT | 149 |
|  |  |  | UPq-Mut-F | CTGTCCTCGGCACGGTGATTTGGTGGGAGGAGAGCGTGTGAGGCCTTACTTCC |  |
|  |  |  | UPq-R | ACCCCCAAGTACAGCCAGGTC |  |
|  |  |  | Intermediate | TCACCGCATGCGTC |  |
| APC | rs35305379 | TTTA>TTTTA | UPq-WT-F | CTGTCCTCGGCACGCAGGGAAGGTGGTAGGTGTCTGTGATCCATTACTAGAGAAGTTTA | 239 |
|  |  |  | UPq-Mut-F | CTGTCCTCGGCACGGTGATTTGGTGGGAGGAGTCTGTGATCCATTACTAGAGAAGTTTT |  |
|  |  |  | UPq-R | AAACTAGAGAAATAAAGTGAATAAGTGGTGA |  |
|  |  |  | Intermediate | CTTTTATTTTTTAATTCTACACAG |  |
| APC | rs34481414 | ACTACAAT>ACAAT | UPq-WT-F | CTGTCCTCGGCACGCAGGGAAGGTGGTAGGTGAATCATCAGGGAATACAAATTAAAACTAC | 210 |
|  |  |  | UPq-Mut-F | CTGTCCTCGGCACGGTGATTTGGTGGGAGGAGAATCATCAGGGAATACAAATTAAAACAAT |  |
|  |  |  | UPq-R | AAAACACTGCCAAATGTTGTCCA |  |
|  |  |  | Intermediate | GCTGCGTGATGATGAA |  |
| GAPDH |  |  | UPq-WT-F | CTGTCCTCGGCACGCAGGGAAGGTGGTAGGTGTGTTGCAACCGGGAAGGAAAT | 231 |
|  |  |  | UPq-Mut-F | CTGTCCTCGGCACGGTGATTTGGTGGGAGGAGTGTTGCAACCGGGAAGGAAAT |  |
|  |  |  | UPq-R | CCTCCATAAACCCACTTCTTTGATT |  |
|  |  |  | Intermediate | CTAACGGCTGCCCATT |  |
| UPq: UPIP-qPCR; WT: wild type; Mut: mutant; Red colored letters are allele specific bases. | | | | | |

| **Table S3** Communal Primers and Probes | |
| --- | --- |
| Name | Sequence (5'-3') |
| FAM universal connector | CTGTCCTCGGCACGCAGGGAAGGTGGTAGGTG |
| HEX universal connector | CTGTCCTCGGCACGGTGATTTGGTGGGAGGAG |
| FAM universal probe | FAM-CACCTACCACCTTCCCTG-BHQ1 |
| HEX universal probe | HEX-CTCCTCCCACCAAATCAC-BHQ1 |
| Universal primer | CTGTCCTCGGCACG |
| FAM-only probe | FAM-CAGGGAAGGTGGTAGGTG |
| HEX-only probe | HEX-AGTGATTTGGTGGGAGGAG |
| Universal reverse connector/primer | TGGGAGCTGAGGGCGA |

| **Table S4** Different Intermediate Primers for ALDH2 rs671 | |
| --- | --- |
| Name | Sequence (5'-3') |
| ALDH2-Inter0 | GGCTGCAGGCATACACT |
| ALDH2-Inter30 | GGAGTGGCCGGGAG |
| ALDH2-Inter60 | CCCAGTCACCCTTTGGT |
| ALDH2-Inter90 | ATTACAGGGTCAACTGCTAT |
| ALDH2-Inter120 | CAAGAGTGATTTCTGCAAT |

| **Table S5** Primers and Probes for TaqMan Probe-qPCR | | |
| --- | --- | --- |
| Gene | Rs. No | Sequence (5'-3') |
| ALDH2 | rs671 | F: CGGGAGTTGGGCGAGTAC |
|  |  | R: TCCGAGCCACCAGCAGAC |
|  |  | Probe1: FAM-CAGGCATACACTGAAGTGAAAAC-MGB |
|  |  | Probe2: HEX-CAGGCATACACTAAAGTGAAAAC-MGB |
| CYP2C9 | rs1057910 | F: CACATGCCCTACACAGATGCT |
|  |  | R: GGAGAAACAAACTTACCTTGGGA |
|  |  | Probe1: FAM-AGGTCCAGAGATACATTGACCTT-MGB |
|  |  | Probe2: HEX-AGGTCCAGAGATACCTTGACCTT-MGB |
| VKORC1 | rs9923231 | F: CAAAATGCTAGGATTATAGGCGT |
|  |  | R: GGAAGTCAAGCAAGAGAAGACC |
|  |  | Probe1: FAM-ACCGCACCCGGCCAAT-MGB |
|  |  | Probe2: HEX-CACCGCACCTGGCCAAT-MGB |
| MTHFR | rs1801131 | F: TCTACCTGAAGAGCAAGTCCC |
|  |  | R: CACTCCAGCATCACTCACTTTG |
|  |  | Probe1: FAM-TGACCAGTGAAGAAAGTGTCTTT-MGB |
|  |  | Probe2: HEX-TGACCAGTGAAGCAAGTGTCTTT-MGB |
| MTHFR | rs1801133 | F: CCTCTCCTGACTGTCATCCCTA |
|  |  | R: GATGCCCATGTCGGTGCA |
|  |  | Probe1: FAM-TGCGGGAGCCGATTTCAT-MGB |
|  |  | Probe2: HEX-TGCGGGAGTCGATTTCAT-MGB |
| MTRR | rs1801394 | F: GCCTTGAAGTGATGAGGAGGT |
|  |  | R: TTCAAAGCACAAAACGGTAAAA |
|  |  | Probe1: FAM-CAGAAGAAATATGTGAGCAAGC-MGB |
|  |  | Probe2: HEX-CAGAAGAAATGTGTGAGCAAGC-MGB |

| **Table S6** Primers for KASP | | |
| --- | --- | --- |
| Gene | Rs. No | Sequence (5'-3') |
| ALDH2 | rs671 | F1: GAAGGTGACCAAGTTCATGCTCCCACACTCACAGTTTTCAATTC |
|  |  | F2: GAAGGTCGGAGTCAACGGATTCCCACACTCACAGTTTTCAATTT |
|  |  | R: CCTGGGAGTGTAACCCATAACC |
| CYP2C9 | rs1057910 | F1: GAAGGTGACCAAGTTCATGCTAGTGCACGAGGTCCAGAGATACA |
|  |  | F2: GAAGGTCGGAGTCAACGGATTAGTGCACGAGGTCCAGTGATACC |
|  |  | R: TAGGAGAAACAAACTTACCTTGGGA |
| VKORC1 | rs9923231 | F1: GAAGGTGACCAAGTTCATGCTAGCGTGAGCCACCGCTCCC |
|  |  | F2: GAAGGTCGGAGTCAACGGATTAGCGTGAGCCACCGAACCT |
|  |  | R: TGGTGACCATTATTCTGTCTACCACACT |
| MTHFR | rs1801131 | F1: GAAGGTGACCAAGTTCATGCTAAGGAGGAGCTGACCAGTGAAGA |
|  |  | F2: GAAGGTCGGAGTCAACGGATTTTGGAGGAGCTGACCAGTTAAGC |
|  |  | R: GGTCCCCACTCCAGCATCACT |
| MTHFR | rs1801133 | F1: GAAGGTGACCAAGTTCATGCTTGAGAAGGTGTCTGCGGAAGC |
|  |  | F2: GAAGGTCGGAGTCAACGGATTTGAGAAGGTGTCTGCGGGTGT |
|  |  | R: GGGAAGAACTCAGCGAACTCAGC |
| MTRR | rs1801394 | F1: GAAGGTGACCAAGTTCATGCTCATGTACCACAGCTTGCTCTCAT |
|  |  | F2: GAAGGTCGGAGTCAACGGATTCATGTACCACAGCTTGCTAACAC |
|  |  | R: CCATTTTTCAGTTTCACTGTTACATGC |

| **Table S7** Minor allele frequency (MAF) analysis of each SNP | | | | | | | |
| --- | --- | --- | --- | --- | --- | --- | --- |
| Rs. No | MAF of East Asian | | | |  | MAF of Chinese Han | |
|  | ALFA | | 1000 Genomes | |  | 224 Samples (UPIP-qPCR) | |
| rs671 | G=0.7821 | A=0.2179 | G=0.8264 | A=0.1736 |  | G=0.8013 | A=0.1987 |
| rs1057910 | A=0.9564 | C=0.0436 | A=0.9663 | C=0.0337 |  | A=0.9241 | C=0.0759 |
| rs9923231 | C=0.095 | T=0.905 | C=0.1151 | T=0.8849 |  | C=0.0804 | T=0.9196 |
| rs1801131 | T=0.786 | G=0.214 | T=0.7808 | G=0.2192 |  | T=0.8304 | G=0.1696 |
| rs1801133 | G=0.6137 | A=0.3863 | G=0.7044 | A=0.2956 |  | G=0.6518 | A=0.3482 |
| rs1801394 | A=0.7270 | G=0.2730 | A=0.7371 | G=0.2629 |  | A=0.3527 | G=0.6473 |
| ALFA: Allele Frequency Aggregator; | | | | | | | |

| **Table S8** Primers for Preparation of DNA Standards Containing Specific SNPs | | | | | |
| --- | --- | --- | --- | --- | --- |
| Gene | Rs. No. | Variation | Primer type | Primer sequence (5'-3') | Product (bp) |
| ALDH2 | rs671 | G>A | Control-WT-F | CAGGTCCCACACTCACAGTTTTCACTTCAGTGT | 191 |
|  |  |  | Control-Mut-F | CAGGTCCCACACTCACAGTTTTCACTTTAGTGT |  |
|  |  |  | Control-R | CCTGGGAGTGTAACCCATAACC |  |
| CYP2C9 | rs1057910 | A>C | Control-WT-F | CTGTGGTGCACGAGGTCCAGAGATACATTGAC | 119 |
|  |  |  | Control-Mut-F | CTGTGGTGCACGAGGTCCAGTGATACCTTGAC |  |
|  |  |  | Control-R | TAGGAGAAACAAACTTACCTTGGGA |  |
| VKORC1 | rs9923231 | C>T | Control-WT-F | TATAGGCGTGAGCCACCGCACCCGGCCA | 220 |
|  |  |  | Control-Mut-F | TATAGGCGTGAGCCACCGCACCTGGCCA |  |
|  |  |  | Control-R | TGGTGACCATTATTCTGTCTACCACACT |  |
| ABCB1 | rs10234411 | A>T | Control-WT-F | TTCAGAAGGCATAGTACCAGCTCCTCTTTGTAAT | 210 |
|  |  |  | Control-Mut-F | TTCAGAAGGCATAGTACCAGCTCCTCTTAGTAAT |  |
|  |  |  | Control-R | TGCAATCTCAGCTCTGCAACCT |  |
| ADD1 | rs4961 | G>T | Control-WT-F | CGGGGCGACGAAGCTTCCGAGGAAGGGCAG | 176 |
|  |  |  | Control-Mut-F | CGGGGCGACGAAGCTTCCGACGAATGGCAG |  |
|  |  |  | Control-R | TGAACAGTAAGGTAGGCACAGACCA |  |
| ADRB1 | rs1801253 | G>C | Control-WT-F | GCCCCGACTTCCGCAAGGCCTTCCAGGGACTG | 77 |
|  |  |  | Control-Mut-F | GCCCCGACTTCCGCAAGGCCTTCCAGCGACTG |  |
|  |  |  | Control-R | TCTCCGTGGGTCGCGTGGC |  |
| MTHFR | rs1801131 | A>C | Control-WT-F | TGGGGGGAGGAGCTGACCAGTGAAGAAAGTG | 111 |
|  |  |  | Control-Mut-F | TGGGGGGAGGAGCTGACCAGTTAAGCAAGTG |  |
|  |  |  | Control-R | GGTCCCCACTCCAGCATCACT |  |
| MTHFR | rs1801133 | C>T | Control-WT-F | TGAAGGAGAAGGTGTCTGCGGGAGCCGATT | 249 |
|  |  |  | Control-Mut-F | TGAAGGAGAAGGTGTCTGCGGGAGTCGATT |  |
|  |  |  | Control-R | GGGAAGAACTCAGCGAACTCAGC |  |
| MTRR | rs1801394 | A>G | Control-WT-F | AAATCCATGTACCACAGCTTGCTCACATATTTC | 129 |
|  |  |  | Control-Mut-F | AAATCCATGTACCACAGCTTGCTCACACATTTC |  |
|  |  |  | Control-R | CCATTTTTCAGTTTCACTGTTACATGC |  |
| ABCB1 | rs1045642 | T>C | Control-WT-F | GCCGGGTGGTGTCACAGGAAGAGATTGTGAG | 164 |
|  |  |  | Control-Mut-F | GCCGGGTGGTGTCACAGGAAGAGATCGTGAG |  |
|  |  |  | Control-R | GGAGACCAGCCCCTTATAAATCAA |  |
| DPYD | rs3918290 | G>A | Control-WT-F | AACTAAAGGCTGACTTTCCAGACAACGTAAGT | 159 |
|  |  |  | Control-Mut-F | AACTAAAGGCTGACTTTCCAGACAACATAAGT |  |
|  |  |  | Control-R | ATCAGCAAAGCAACTGGCAGATT |  |
| DPYD | rs55886062 | A>C | Control-WT-F | CTCCAGCCACCAGCACATCAATGATTCGAA | 252 |
|  |  |  | Control-Mut-F | CTCCAGCCACCAGCACATCAATGAGTCGAA |  |
|  |  |  | Control-R | GGGACAGAAAGGAAGGAAAGAAACT |  |
| GSTP1 | rs1695 | A>G | Control-WT-F | GTGGAGGACCTCCGCTGCAAATACATCTCC | 139 |
|  |  |  | Control-Mut-F | GTGGAGGACCTCCGCTGCAAATACGTCTCC |  |
|  |  |  | Control-R | CCAAGCCACCTGAGGGGTAAG |  |
| XRCC1 | rs25487 | A>G | Control-WT-F | GGTTGGCGTGTGAGGCCTTACCTCTGGGAG | 122 |
|  |  |  | Control-Mut-F | GGTTGGCGTGTGAGGCCTTACCTCCGGGAG |  |
|  |  |  | Control-R | ACCCCCAAGTACAGCCAGGTC |  |
| APC | rs35305379 | TTTA>TTTTA | Control-WT-F | TCTGTGATCCATTACTAGAGAAGTTTA | 212 |
|  |  |  | Control-Mut-F | TCTGTGATCCATTACTAGAGAAGTTTT |  |
|  |  |  | Control-R | AAACTAGAGAAATAAAGTGAATAAGTGGTGA |  |
| APC | rs34481414 | ACTACAAT>ACAAT | Control-WT-F | TCATTAATCATCAGGGAATACAAATTAAAACTACAATGA | 183 |
|  |  |  | Control-Mut-F | TCATTAATCATCAGGGAATACAAATTAAAACAATGAGAT |  |
|  |  |  | Control-R | AAAACACTGCCAAATGTTGTCCA |  |
| GAPDH |  |  | Control-WT-F | CTGAGTCATGGGTAGTTGGAAAAGGACATTTCC | 129 |
|  |  |  | Control-R | GGTCGGGTCAACGCTAGGCTG |  |

| **Table S9** Amino Modified Probes for Microarrays | | | |
| --- | --- | --- | --- |
| No. | Name | Rs. No. | Sequence (5'-3') |
| 1 | FAM-Cont |  | NH2-ttttttttttttttCACCTACCACCTTCCCTG |
| 2 | HEX-Cont |  | NH2-ttttttttttttttCTCCTCCCACCAAATCACT |
| 3 | ALDH2 | rs671 | NH2-ttttttttttttttGCTGCAGGCATACACTT |
| 4 | CYP2C9 | rs1057910 | NH2-ttttttttttttttGTGGGGAGAAGGTCAA |
| 5 | VKORC1 | rs9923231 | NH2-ttttttttttttttGACCTGAAAAACAACCAT |
| 6 | ABCB1 | rs10234411 | NH2-ttttttttttttttGTTTGTATTAAAACAACAATTAC |
| 7 | ADD1 | rs4961 | NH2-ttttttttttttttGGACTGCTTCCATTCT |
| 8 | ADRB1 | rs1801253 | NH2-ttttttttttttttGCGCAGCAGAGCAGT |
| 9 | MTHFR | rs1801131 | NH2-ttttttttttttttGGTTCTCCCGAGAGGT |
| 10 | MTHFR | rs1801133 | NH2-ttttttttttttttAGCCTCAAAGAAAAGCT |
| 11 | MTRR | rs1801394 | NH2-ttttttttttttttAGGCCATCGCAGAAGA |
| 12 | ABCB1 | rs1045642 | NH2-ttttttttttttttTTGGCCTCCTTTGC |
| 13 | DPYD | rs3918290 | NH2-ttttttttttttttTTGTTTTAGATGTTAAATCACA |
| 14 | DPYD | rs55886062 | NH2-ttttttttttttttCTTCAAAAGCTCTTCGA |
| 15 | GSTP1 | rs1695 | NH2-ttttttttttttttTTGGTGTAGATGAGGGAGA |
| 16 | XRCC1 | rs25487 | NH2-ttttttttttttttTCACCGCATGCGTC |
| 17 | APC | rs35305379 | NH2-ttttttttttttttCTTTTATTTTTTAATTCTACACAG |
| 18 | APC | rs34481414 | NH2-ttttttttttttttGCTGCGTGATGATGAA |
| 19 | GAPDH |  | NH2-ttttttttttttttCTAACGGCTGCCCATT |
| 20 | Negative-Cont |  | NH2-tttttttttttttt |

| **Table S10** Genotyping Results of Sample No.1 by Multiplex UPIP-qPCR | | | | |
| --- | --- | --- | --- | --- |
| Gene | Rs. No. | Variation | Genotype of Sample No.1 | |
|  |  |  | Multiplex UPIP-qPCR | Sanger |
| ALDH2 | rs671 | G>A | GG | GG |
| CYP2C9 | rs1057910 | A>C | AC | AC |
| VKORC1 | rs9923231 | C>T | TT | TT |
| ABCB1 | rs10234411 | A>T | TT | TT |
| ADD1 | rs4961 | G>T | GT | GT |
| ADRB1 | rs1801253 | G>C | CC | CC |
| MTHFR | rs1801131 | A>C | AC | AC |
| MTHFR | rs1801133 | C>T | CT | CT |
| MTRR | rs1801394 | A>G | AA | AA |
| ABCB1 | rs1045642 | T>C | TC | TC |
| DPYD | rs3918290 | G>A | GG | GG |
| DPYD | rs55886062 | A>C | AA | AA |
| GSTP1 | rs1695 | A>G | AA | AA |
| XRCC1 | rs25487 | A>G | AG | AG |
| APC | rs35305379 | TTTA>TTTTA | TTA/TTTA | TTA/TTTA |
| APC | rs34481414 | ACTACAAT>ACAAT | ACTACAAT/ACAAT | ACTACAAT/ACAAT |

| **Table S11** Genotyping Results of Sample No.1 & 2 by Microarrays | | | | | | |
| --- | --- | --- | --- | --- | --- | --- |
| Gene | Rs. No. | Variation | Genotype of Sample No.1 | | Genotype of Sample No.2 | |
|  |  |  | Microarray | Sanger | Microarray | Sanger |
| ALDH2 | rs671 | G>A | GG | GG | AA | AA |
| CYP2C9 | rs1057910 | A>C | AC | AC | AA | AA |
| VKORC1 | rs9923231 | C>T | TT | TT | TT | TT |
| ABCB1 | rs10234411 | A>T | TT | TT | AA | AA |
| ADD1 | rs4961 | G>T | GT | GT | GG | GG |
| ADRB1 | rs1801253 | G>C | CC | CC | GC | GC |
| MTHFR | rs1801131 | A>C | AC | AC | AA | AA |
| MTHFR | rs1801133 | C>T | CT | CT | CC | CC |
| MTRR | rs1801394 | A>G | AA | AA | AA | AA |
| ABCB1 | rs1045642 | T>C | TC | TC | CC | CC |
| DPYD | rs3918290 | G>A | GG | GG | GG | GG |
| DPYD | rs55886062 | A>C | AA | AA | AA | AA |
| GSTP1 | rs1695 | A>G | AA | AA | GG | GG |
| XRCC1 | rs25487 | A>G | AG | AG | AG | AG |
| APC | rs35305379 | TTTA>TTTTA | TTA/TTTA | TTA/TTTA | TTA/TTTA | TTA/TTTA |
| APC | rs34481414 | ACTACAAT>ACAAT | ACTACAAT/ACAAT | ACTACAAT/ACAAT | ACTACAAT/ACAAT | ACTACAAT/ACAAT |
